# Tuning endothelial barrier permeability with ultrasound: a pulse-length-dependent interplay between bubble dynamics and cellular bioeffects

**DOI:** 10.1016/j.ultsonch.2026.107851

**Published:** 2026-04-12

**Authors:** Chaofeng Qiao, Siyu Luo, Zhihui Liu, Yingxuan Bu, Yicong Cai, Zhuoyan Liu, Liying Wang, Claus Dieter Ohl, Fenfang Li

**Affiliations:** a School of Basic Medical Sciences, Beihua University, Jilin City, China; b Institute of Molecular Physiology, Shenzhen Bay Laboratory, Shenzhen, China; c School of Biology and Biological Engineering, South China University of Technology, Guangzhou 510006, China; d Institute of Physics, Otto-von-Guericke University Magdeburg, Germany

**Keywords:** Ultrasound pulses, Microbubble cavitation dynamics, Vessel-mimicking microchannels, Membrane perforation, Ca^2+^ signalling, Reactive oxygen species, Trans-endothelial molecular transport

## Abstract

Ultrasound mediated microbubble cavitation holds great potential for non-invasive and targeted drug delivery. However, the interplay between acoustic parameters, bubble dynamics, and resulting cellular responses remains unclear, hindering the safety improvement and optimization of the technique. This study examined the effects of ultrasound pulse sequences on microbubble dynamics and bioeffects in endothelial monolayer using an acoustically coupled vessel-mimicking microchannels, where focused ultrasound exposure and concurrent recording of Ca^2+^ signalling and membrane perforation were performed at flow conditions. A reduction of the total treatment time from 60 to 10 s avoided cell detachment. Microbubbles demonstrated brief oscillation and displacement under each of the 10 consecutive bursts of 40  µs short pulses with 1 ms interval while more intense bubble clustering, coalescence and displacement were observed under one continuous long pulse that lasted for around 9 ms. 10 s long pulse generated higher percentage and larger extent of cell membrane poration whereas short pulse induced wider spreading and larger Ca^2+^ signalling across the cell population. Reactive oxygen species, extracellular Ca^2+^ influx through mechanosensitive channels and internal Ca^2+^ release were found critical in mediating Ca^2+^ responses in short pulse condition. Further transwell experiments revealed that both pulse modes enhanced transport of 10  kDa FITC-dextran while a longer treatment of 60 s improved delivery efficiency for larger FITC-dextran of 40 kDa. These findings highlight the importance of pulse modes and total treatment time in tailoring Ca^2+^ signalling mediated paracellular transport and sonoporation mediated transcellular transport, offering insights for optimizing ultrasound parameters for therapeutic drug delivery.

## 1 Introduction

The interaction between ultrasound, microbubbles, and biological barriers represents a quintessential sonomechanical and sonochemical process at the bio-interface. When driven by acoustic fields, microbubbles undergo cavitation, a phenomenon encompassing either inertial expansion and collapse, or stable oscillation, and the generation of localized physical and chemical conditions [1], [2]. These include acoustic microstreaming and microjets that create shear stresses [3], [4], [5], [6], and the production of reactive oxygen species (ROS) [7], [8], which collectively modulate cellular behavior and barrier function. The endothelial barrier is a critical regulator of molecular exchange between the bloodstream and underlying tissues. In the brain, this structure forms the blood–brain barrier (BBB), which tightly controls cerebral homeostasis but also impedes the delivery of therapeutic agents to the central nervous system [9], [10]. Focused ultrasound combined with microbubbles has emerged as a promising strategy that harnesses cavitation effects to enhance drug delivery [11], allowing transient and local opening of the BBB for treatment of brain cancers [12] and neurological disorders [13], [14]. A critical and underexplored frontier, however, lies in deciphering how the temporal profile of different acoustic pulse drives specific and distinct cavitation dynamics. These effects, in turn, could differentially influence the endothelial barrier permeability, e.g., by activating specific intracellular signaling cascades that ultimately dictate biological outcomes.

Central among these cascades is Ca^2+^ signaling, a ubiquitous second messenger in cellular signal transduction of mechanical and chemical stimuli [15]. In endothelial cells, Ca^2+^ influx through mechanosensitive channels or release from intracellular stores can trigger actomyosin contraction and tight junction rearrangement, leading to reversible increases in paracellular permeability [7], [16]. Ca^2+^ signaling can promote exocytosis for cell plasma membrane repair after sonoporation [17] while overload of intracellular Ca^2+^ is toxic to cells, leading to cell apoptosis even after membrane pores have resealed [4], [18]. While the mechanical forces from cavitation are known to cause membrane poration (sonoporation) or direct cell detachment [19], [20], [21], the concomitant sonochemical and biochemical signaling events, particularly ROS generation and cellular Ca^2+^ signaling are hypothesized to influence whether the cellular response is adaptable and reversible, or irreversible and lethal [4], [22], [23], [24].

Long-pulse (LP) ultrasound sequences are employed in most preclinical and clinical studies [25], [26], which are effective in disrupting the BBB but have been associated with adverse effects such as erythrocyte extravasation [27], edema [28], and inflammation [29]. These outcomes suggest that prolonged acoustic exposure may exceed the endothelial capacity for homeostatic recovery. In contrast, emerging short-pulse (SP) ultrasound protocols, characterized by brief, repetitive bursts, deliver significantly less energy and have shown promise in achieving more uniform and reversible barrier opening with minimal tissue damage [30], [31], [32], [33]. However, the explicit link between ultrasound pulse parameters, microbubble cavitation dynamics, the resulting sonoporation and Ca^2+^ signaling characteristics and their downstream consequences on endothelial barrier permeability remains incompletely understood, especially under physiological flow conditions [23], [34], [35]. For instance, while differences in microbubble cavitation behavior between pulse lengths have been documented, direct high-speed optical observation is limited and lacks spatiotemporal resolution on the single bubble scale [36], [37]. It is unclear how these distinct profiles of cavitation behavior translate into differential patterns, e.g., amplitude, kinetics and spatial uniformity of intracellular Ca^2+^ signaling and membrane poration in endothelial monolayers, and whether sonochemical generated ROS and mechanically activated ion channels contributed to initiating and shaping the Ca^2+^ signals. This gap limits our ability to rationally design ultrasound protocols that maximize therapeutic efficacy while minimizing size effects.

In this study, we investigate this question using a vessel-mimicking microfluidic platform that permits high-resolution observation of cavitation dynamics and real-time live-cell fluorescence imaging of Ca^2+^ signaling and membrane poration. We focus on contrasting clinically relevant LP and SP ultrasound at conditions that avoid cell detachment by reducing the total treatment time from 60 s to 10 s. In this study, Long Pulse (LP) ultrasound was applied as one continuous burst per second, with each burst lasting 9.09  ms, repeated over a total treatment time of up to tens of seconds. In contrast, Short Pulse (SP) ultrasound also consisted of one burst per second, but each burst comprised 10 sub-pulses with a duration of 40  µs each, separated by 1  ms intervals, for the same total treatment duration as the LP mode. Our integrated experimental approach revealed that LP ultrasound triggered more intense bubble displacement, clustering, and coalescence, leading to higher percentage and larger extent of cell membrane poration and heterogeneous Ca^2+^ transients. In contrast, SP ultrasound induced brief oscillation and displacement of bubbles under each of the 40  µs short pulse exposure, resulting in a wider spreading, more uniform and larger Ca^2+^ elevation coupled with less membrane poration. Pharmacological interrogation established ROS as a critical sonochemical mediator linking cavitation to Ca^2+^ response for SP exposure condition. Further transwell experiments revealed comparable capability of both pulse conditions for molecular transport of 10  kDa and 40 kDa FITC-dextran across endothelial barrier at 10 and 60 s total treatment time. By bridging the fields of acoustic cavitation physics, sonochemistry, and cell signaling biology, this work advances the fundamental understanding of how acoustic energy is converted into biochemical cellular commands. It underscores that the therapeutic window of ultrasound-mediated barrier opening is not only defined by cavitation activity, but may also by the nature of the signaling cascades it activates. Our results thus contribute to the foundation for designing smarter, signaling-aware ultrasonic stimuli for safer and more effective drug delivery.

## 2 Materials and methods

### 2.1 Design and fabrication of vessel-mimicking microchannels

**Fig. 1:**
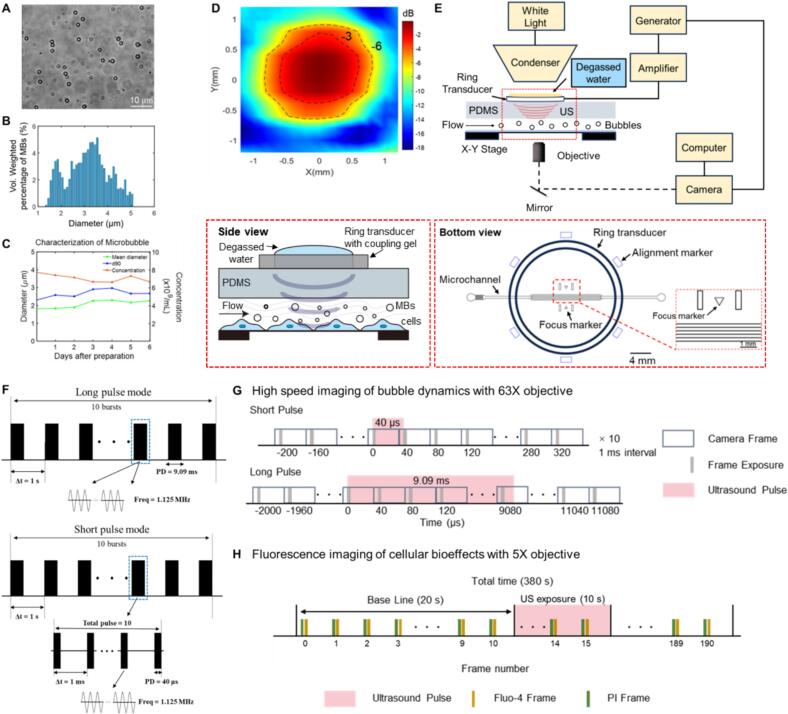
**Experimental setup and design**. (A) The morphology, (B) Size distribution and (C) Stability of home-made microbubbles with a lipid shell and perfluoropropane core, d90: the size below which 90% of the particles fall within. (D) Characterization of the acoustic field produced by the 1.125 MHz ring ultrasound transducer in the X-Y focal plan. The acoustic pressure is shown in dB relative to the peak value. (E) Experimental setup for ultrasound stimulation, imaging of bubble dynamics and cellular bioeffects inside three parallel microchannels on an inverted microscope. The insets show the enlarged side view and bottom view of the alignment of the ring ultrasound transducer with the microchannels outlined by the red dashed box. (F) Schematic of the ultrasound waveforms used in the experiments: long pulse and rapid short pulse mode. (G) Recording sequences for high-speed imaging of the bubble dynamics and synchronization with ultrasound exposure in short and long pulse mode. (H) Concurrent fluorescent imaging of membrane poration (PI) and calcium signaling (Fluo-4), and synchronization with 10 s ultrasound exposure in short and long pulse mode with a 20 s baseline recording.

Microchannels mimicking blood vessels were designed using AutoCAD, with the corresponding SU-8 master mold fabricated using standard soft lithography techniques. A 10:1 mixture of polydimethylsiloxane (PDMS, Sylgard 184 Silicone Elastomer Kit, Dow Corning (Dowsil), Midland, MI, USA) was poured onto the mold, cured at 60 °C for 4 h, and subsequently bonded to #1 cover glass slides (25 × 50 mm^2^, 0.13–0.17 mm thick) immediately after a 50-second plasma treatment (Zepto one, Diener, Ebhausen, Germany). The resulting microfluidic chips contains three separate channels, each measuring 200 µm wide, 100 µm high, and 17000 µm long. To ensure accurate alignment of the focus of the ring ultrasound transducer with the microchannels, marker patterns were incorporated into the design (Fig. 1A). The height of the PDMS layer was maintained at 3 mm to position the microchannels at the ultrasound beam’s focal point along the z-axis. The transmission loss introduced by the thin glass substrate is 1.24dB (Fig. S1 and Fig. S2). Therefore, approximately 86.7% of the acoustic pressure amplitude is retained past the glass substrate.

### 2.2 Preparation and characterization of microbubbles

Home-made microbubbles were prepared as previously described [38]. Their structure comprised a lipid shell encapsulating a perfluoropropane (C_3_F_8_) gas core. The lipid formulation consisted of 1,2-distearoyl-*sn*-glycero-3-phosphocholine (DSPC) and N-(carbonyl-methoxypolyethylene glycol-2000)-1,2-distearoyl-*sn*-glycero-3-phosphoethanolamine(DSPE-PEG2000) (Lipoid, Ludwigshafen, Germany) in a 9:1 M ratio. After the synthesis process, the microbubbles were diluted with Isoton II solution, and their concentration and size distribution were measured using a Coulter Counter Multisizer IV (Beckman Coulter Inc., USA). Their morphology was then examined under a microscope (BX-53, Olympus Corporation, Japan). For volume-weighted bubble size distribution, each measured bubble diameter was defined as D_i_, the individual bubble volume was calculated as Vi = πD_i_^3^/6. The volume fraction for each bin of size was computed as the ratio of the total volume of bubbles within that bin to the total volume of all measured bubbles.

### 2.3 Cell culture, preparations and handling

Murine brain microvascular endothelial cells (bEnd.3) were maintained in DMEM (Gibco, C11995500BT) supplemented with 10% heat-inactivated fetal bovine serum and 1% penicillin–streptomycin, cultured at 37 °C in a humidified atmosphere containing 5% CO_2_. Cells between passages 5 and 12 were utilized for this study.

Before experiments, cells at approximately 90% confluency were detached using trypsin, resuspended in prewarmed (37 °C) culture medium at a concentration of 1 × 10^7^ cells/mL, and loaded into the microchannels using a sterilized 1 mL syringe. Prior to cell seeding, the microchannels were perfused with 1X PBS for 5 min, followed by coating with 50 µg/mL fibronectin (Roche, 10838039001) for 15 min at 37 °C, and rinsing with 1X DPBS (Gibco, 14040133, with Ca^2+^). Once cells were introduced into the microchannels and reached 60–70% density, the inlet and outlet tubing were clamped, and the device was incubated under static conditions for 30 min to promote cell adhesion. Subsequently, the microchannels were examined every 30 min to assess cell viability and confluency, and fresh culture medium was infused using a 1 mL syringe at 2 µL/min with a syringe pump (KDS, R462) to replenish nutrients and remove metabolic waste. After approximately 6 h when cells form a stable monolayer, the microchannels were perfused with DMEM containing 5 µg/mL Hoechst 33,342 (biosharp, BL803A) and 6 µM Fluo4-AM (Invitrogen, F14201) to label cell nuclei and monitor intracellular Ca^2+^ fluctuations, respectively. Following a 15-minute incubation at 37 °C in darkness, excess dyes were gently washed out by 1X DPBS at 2 μL/min for 1 min. The microfluidic chip was then placed on the microscope stage, where 1X DPBS (with Ca^2+^) containing microbubbles (diluted 1:20 v/v) and propidium iodide (PI, Thermo Fisher Scientific, P21493) at a final concentration of 100 µg/mL was perfused at 75 µL/min.

### 2.4 Setup for recording bubble dynamics or cellular bioeffects in microchannels

For bubble dynamics, the home-made microbubbles were diluted 1:20 (v/v) in 1X DPBS and delivered into the microchannels at a flow rate of 75 µL/min using the syringe pump on an inverted microscope (Zeiss, Axio Observer 7), as shown in Fig. 1. Sonication was performed with a custom-built, ring-shaped ultrasound transducer operating at 1.125 MHz and driven by a 50-dB power amplifier (2100 L, Electronics & Innovation, USA). This transducer was positioned on top of the PDMS chip with high-vacuum grease gel (Dow Corning) and aligned to the microchannels with reference markers (Fig. 1). The driving ultrasound signals were produced by function generator (DG972, RIGOL, China). Two distinct ultrasound protocols were applied: a long-pulse scheme (one pulse per second, each pulse consisting of a 9.09 ms continuous wave, total 10 pulses) and a rapid short-pulse scheme (one pulse per second, total 10 pulses, each pulse containing 10 sub-pulses with a 1 ms interval between sub-pulses and a sub-pulse duration of 40 µs). Therefore, the short-pulse sequence has a smaller integral exposure time and a reduced total acoustic energy compared to the long-pulse mode. Bubble dynamics was recorded with a 63 × objective (LD PN 63×/0.75 Corr) using a high-speed camera (Nova S12, Photron) that was synchronized to the ultrasound pulses (Fig. 1). The high-speed camera was operated at 25,000 frames per second (fps) with an exposure time of 0.66 µs.

For cellular bioeffects studies, the high-speed camera was replaced with a sCMOS camera (EDGE 4.2; PCO) to capture bright-field or fluorescence images of cells through a 5 × microscope objective (N-Achroplan 5×/0.15), allowing simultaneously recording the three parallel microchannels. The sCMOS camera’s ‘exposure out’ signal triggers a digital delay generator (DG535, Stanford Research Systems), enabling the ultrasound system to be activated after 20 s of baseline fluorescence recording. Fluorescence (FL) imaging, with a total duration of 380 s and 100 ms exposure times for concurrent PI and Fluo-4 recording, was performed using µManager (version 2.0; open-source). The software synchronized the Cool LED fluorescence excitation, the microscope’s filter turret, and the PCO camera. This configuration allowed alternating acquisitions of PI and Fluo-4 fluorescence at an interval of approximately 1.1 s. Single-shot bright-field images were acquired before and after the fluorescence imaging to evaluate changes in cell morphology.

### 2.5 Mechanistic studies of cellular Ca^2+^ response induced by rapid short pulse ultrasound

The following treatments were performed for mechanistic studies after Fluo-4 AM loading: Before the experiment, cells in the chip were incubated for 15 min in the culture medium with either 3 μM Thapsigargin to deplete internal storage of Ca^2+^, or 10 μM ruthenium red (RR, R2751, Sigma-Aldrich) to block mechanosensitive ion channels. Ascorbic acid was used to quench reactive oxygen species (ROS) generated by the bubble activity. During the experiment, 1X DPBS (with Ca^2+^) containing microbubbles (diluted 1:20 v/v) and 100 µg/mL propidium iodide (PI, Thermo Fisher Scientific, P21493) with either 3 μM thapsigargin or 10 μM RR or 2 mM AA was perfused to the chip inlet at 75 µL/min for 5 min before and throughout sonication and image recording.

### 2.6 Trans-endothelium diffusion analysis in the transwell

Due to geometric constraints, the ring transducer used for the vessel-mimicking microchannel experiments could not be directly applied to the transwell setup. Therefore, we used a commercial single element transducer (1 MHz fundamental frequency, purchased from Hyus Meditec, Shenzhen). To ensure consistency in acoustic exposure across both models, we used a needle hydrophone to calibrate the acoustic fields of both transducers. As shown in Fig. S3, the driving voltages were carefully adjusted so that the peak negative pressures (PNP) at the target planes were identical (0.25 MPa and 0.5 MPa). Moreover, the focal spot sizes were sufficiently large to uniformly cover the regions of interest in both setups. Thus, despite using different transducers, the localized acoustic stimuli were rigorously matched, ensuring comparability of the mechanical effects on cells across all experiments.

#### 2.6.1 Cell culture and barrier formation

2 × 10^5^ bEnd.3 cells were seeded on the underside of a transwell membrane (Corning® Transwell®, 24 mm diameter, 0.4 μm pore polyester). Prior to seeding, the membrane was precoated with 0.1% gelatin solution (Coolaber, SL94002, China) in 1X DPBS (containing Ca^2+^). Following a 2 h incubation for cell attachment, the inserts were transferred to transwell plates. The chambers were filled with 2 mL of DMEM in both the upper and lower compartments, and the model was cultured for 5 days at 37 °C with 5% CO_2_ to form a barrier. The *trans*-endothelial electrical resistance (TEER) were monitored each day using a Millicell ERS-2 V/ohm meter. Subsequent experiments were initiated only after a stable and consistent TEER was achieved.

#### 2.6.2 Quantification of diffused FITC-Dextran with microplate reader

Serial dilutions of 10 and 40 kDa FITC-dextran were prepared to generate standard curves, with fluorescence intensities measured on a microplate reader (Synergy H1, BioTek). For permeability analysis across cell barrier, both chambers of the transwell were washed twice with 1X DPBS. The lower chamber was then filled with 2 mL of 1X DPBS, and the upper chamber with 2 mL of 1X DPBS containing 12.5 μg/mL FITC-Dextran (10 or 40 kDa; Beyotime). Immediately prior to ultrasound treatment, a 200 μL baseline sample was collected from the lower chamber for microplate reader analysis, followed by the addition of 100 μL 4x 10^9^ microbubbles to the lower chamber. After long or short pulse ultrasound treatment, the transwell was returned to the incubator for 1 h, after which a second 200 μL sample was collected from the lower chamber. The FITC-Dextran concentration in all collected samples was determined using a microplate reader and the standard curves to assess the barrier permeability.

### 2.7 Image processing and data analysis

High speed videos of bubble dynamics were imported into MATLAB (The MathWorks, Natick, MA, USA, academic use) to analyze the diameter of individual microbubbles and the total number of bubbles in each image frame. All images were pre-processed with two bilinear interpolation for image upscaling [39]. The centroid and the diameter of individual bubbles in each image frame were then detected by circular Hough transform. And the volume-weighted average bubble diameter was calculated for each image frame: D_v_ = $\frac{\Sigma_{i=1}^{n}V_{i}*D_{i}}{\Sigma_{i=1}^{n}V_{i}}$, Where $D_{i}$ is the diameter of the *i* th bubble, $V_{i}$ is the volume of the *i* th bubble ($V_{i}=4\pi /3{\left(D/2\right)}^{3}$, and *n* is the total number of bubbles in the image frame. To quantify the dynamics of the bubble population, relative bubble diameter change was defined as $\frac{D_{\max}-D_{0}}{D_{0}}$, and the number change was calculated as $\frac{N-N_{0}}{N_{0}}$. Here D_0_ and N_0_ is the mean value of D_v_ and the average number of bubbles over the 200 μs baseline recording before ultrasound, respectively, D_max_ is the maximum D_v_ during the ultrasound exposure, and N is the average number of bubbles over the 200 μs imaging time after ultrasound exposure.

For the analysis of fluorescence images, we obtained the total number of cells and their locations using automatic detection of the cell nuclei with Stardist module of ImageJ 1.54f (NIH, USA) for the Hoechst staining images before ultrasound exposure. Based on the localization of the cell nuclei, we detected the number of PI positive cells before and after ultrasound exposure and the change of PI and Fluo-4 fluorescence intensity.

### 2.8 Statistical analysis

Significant differences were determined by un-paired Student *t*-test for comparison between two groups. One-Way ANOVA was used for comparison of three groups.

## 3 Results

### 3.1 Experimental system and study design

The freshly synthesized microbubbles displayed a polydisperse nature, with a concentration of 7.6 × 10^9^ mL^−1^ and a bimodal nature of the volume-weighted peak distribution, which is characteristic of Definity^TM^ agent[23] (Fig. 1A–B). Volume-weighted size distributions are used here as it has been shown to correlate with microbubble acoustic activity [40], [41]. Ninety percent of the microbubbles had diameters below 2.3 μm (d90)(Fig. 1C), while Martinez et al. reported a d90 of 3.06 μm for microbubbles prepared using the Definity lipid composition[42]. Additionally, the microbubbles in our preparation are less than 10 μm, aligning with Definity's clinical safety profile. Therefore, our microbubbles exhibit size distribution characteristics comparable to Definity. Over 6 days following preparation, the mean diameter increased by 25%, while the concentration decreased by 13.2%, indicating a rather stable population.

We have integrated a custom-built 1.125 MHz ring ultrasound transducer (thickness is 0.126 cm, outer and inner diameter is 1.1 and 0.9 cm, respectively) with an inverted microscope to deliver pulsed ultrasound to target bEnd.3 cell monolayer grown inside vessel-mimicking microchannels. The acoustic pressure output by the ring transducer was measured using a needle hydrophone (Onda, HNR-0500) in a tank with degased water. The focus is at geometric center in the X-Y plane with a −6 dB diameter of around 1.5 mm accounting for the attenuation in PDMS (Fig. 1D) and 3 mm below its emitting surface in Z-direction. The ultrasound ring transducer was carefully aligned to the vessel-mimicking microchannels to actuate bubble activity with the focused ultrasound beam, thus, to stimulate cells under flow conditions on the inverted microscope (Fig. 1E). The hollow and compact structure of the ring transducer doesn’t affect the optical imaging, offers precise alignment relative to the microfluidic device and a high degree of flexibility for the experiments as compared to conventional bulkier ultrasound transducers.

In the experiments, the ultrasound was operated for long (one burst per second with 9.09-ms-long pulses for 10 bursts) or short pulses (10 bursts in total with one burst per second, within each burst, the pulses were emitted at a repetition frequency (PRF) of 1 kHz with a pulse length of 40 µs) mode (Fig. 1F). The peak-negative acoustic pressure was 0.25 or 0.50 MPa after attenuation through PDMS and the total treatment time was 10 s. The effective exposure time of short-pulse ultrasound is 10×10×40μs=4ms whereas for long pulse it is 10×9.09ms=90.9ms. Bubble dynamics were recorded with the 63X objective by high-speed imaging synchronized with the ultrasound pulses (Fig. 1G). Cellular bioeffects were monitored by concurrent fluorescence imaging with propidium iodide (membrane poration indicator) and Fluo-4 (Ca^2+^ signaling indicator) loaded in the cells (Fig. 1H). Fluorescence imaging was first recorded at baseline level for 20 s before the 10 s ultrasound stimulation, followed by a sequence of fluorescence imaging for a total time of around 380 s. Snapshots of the bright field (BF) images and Hoechst fluorescence were captured both immediately before and after the fluorescence recording. As flow rate affects sonoporation of endothelial cells[43], all the cell experiments in the microchannels were performed at a flow rate of 75 ul/min.

**Fig. 2:**
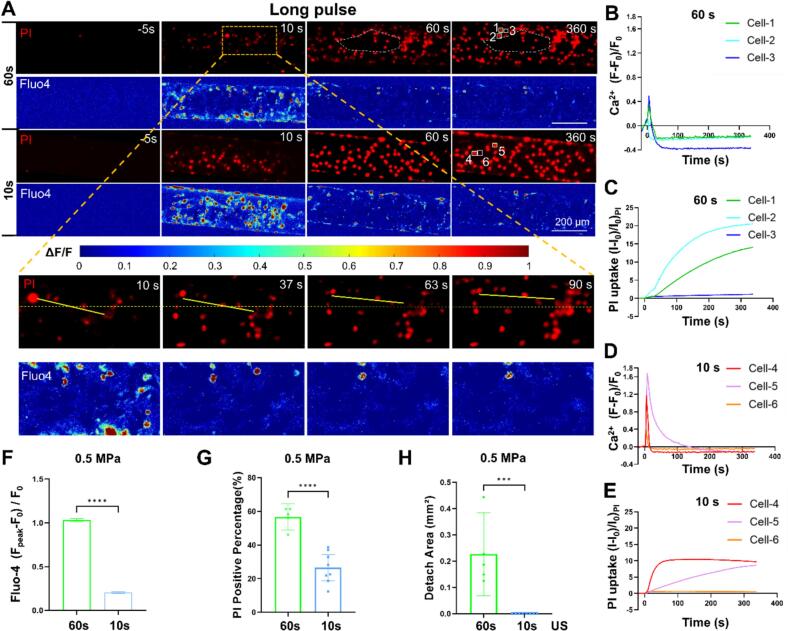
**A comparative analysis of bioeffects induced by 60  s vs. 10  s long-pulse ultrasound stimulation with peak negative acoustic pressure of 0.5  MPa at a flow rate of 75 ul/min.** (A) Image sequences showing PI uptake (red) and Ca^2+^ signaling (Fluo-4, pseudo color) before, during (0–60 s or 0–10 s), and after 0.5 MPa ultrasound exposure at long pulse mode. The inset shows the enlarged view of PI and Fluo-4 fluorescence change from 10 s to 90 s inside the yellow dashed rectangle for 60  s long-pulse ultrasound treatment, demonstrating the movement and detachment of cells. The yellow line in the inset of panel A highlights cellular displacement occurring at the edge of the region where detachment has taken place. Ca^2+^ response and PI uptake (I-I_0_)/I_0_)_PI_ vs. time for exemplary cells labeled with 1,2, and 3 for the 60  s long pulse mode (B-C) and labeled by 4–6 at 10 s long pulse mode (D & E). (F) The peak change of Ca^2+^ transient from individual cells treated with 60  s and 10  s long pulse ultrasound, for *N* = 6275 and *N* = 11300 cells, respectively. Statistical analysis of the percentage of cells showing PI uptake (G) and the total detachment area (H) in each independent microfluidic chip experiment. Here, 5 and 8 repeated and independent microfluidic chip experiments were performed with 60  s and 10  s long pulse ultrasound, respectively. The student *t*-test was used for statistical analysis in F, G, and H panels. Please note that in panel G and H the statistical tests treat chip-level replication as the experimental unit while panel F treats individual cells as independent units.

Our previous work established that prolonged (e.g., 60 s) long pulse ultrasound stimulation causes severe and irreversible cellular damage, including topical cell detachment. To mitigate this, we reduced the ultrasound exposure duration to 10 s. Compared to the 60 s exposure, the 10 s long pulse protocol also resulted in significant cell membrane poration and Ca^2+^ signaling but no discernable cell detachment (Fig. 2A). We analyzed cells labeled 1 to 3 (60 s stimulation) and 4 to 6 (10 s stimulation). The shorter ultrasound stimulation time was associated with a decrease in PI uptake but a more robust Ca^2+^ response, with a higher peak signal than in the 60  s group (Fig. 2B–E). This may be caused by the Ca^2+^ indicator leakage due to excessive membrane poration in cells labeled 1 and 2. A quantitative analysis of the entire cohort revealed that the peak values of Ca^2+^ transient from all individual cells were still significantly larger than in the 60 s group (Fig. 2F). This apparent contradiction is likely caused by the higher percentage of PI positive cells in the 60 s group (Fig. 2G), as our previous studies demonstrated that notable Ca^2+^ response could be evoked by cell membrane poration. Notably, even 10 s long pulse ultrasound stimulation was sufficient to elicit obvious Ca^2+^ signaling in bEnd.3 cells without cell detachment (Fig. 2H) in the vessel-mimicking channel in presence of a flow. Therefore, we conducted further experiments and analysis of bubble dynamics and cellular bioeffects for 10  s long and short pulse conditions.

Please note that the 60 s long pulse condition, which resulted in cell detachment, was deliberately included to establish the upper safety limit of ultrasound exposure. This condition helps to define the boundary between reversible modulation and irreversible damage. Long pulse durations (5–10 ms) are clinically used, and understanding their safety margins—including the transition to damaging regimes—is essential for a rational protocol design. By demonstrating that reducing total treatment time to 10 s eliminates detachment while preserving therapeutic effects, we identify a clinically relevant parameter space that balances efficacy and safety.

### 3.2 Bubble dynamics in microchannels at short pulse and long pulse ultrasound exposures

**Fig. 3:**
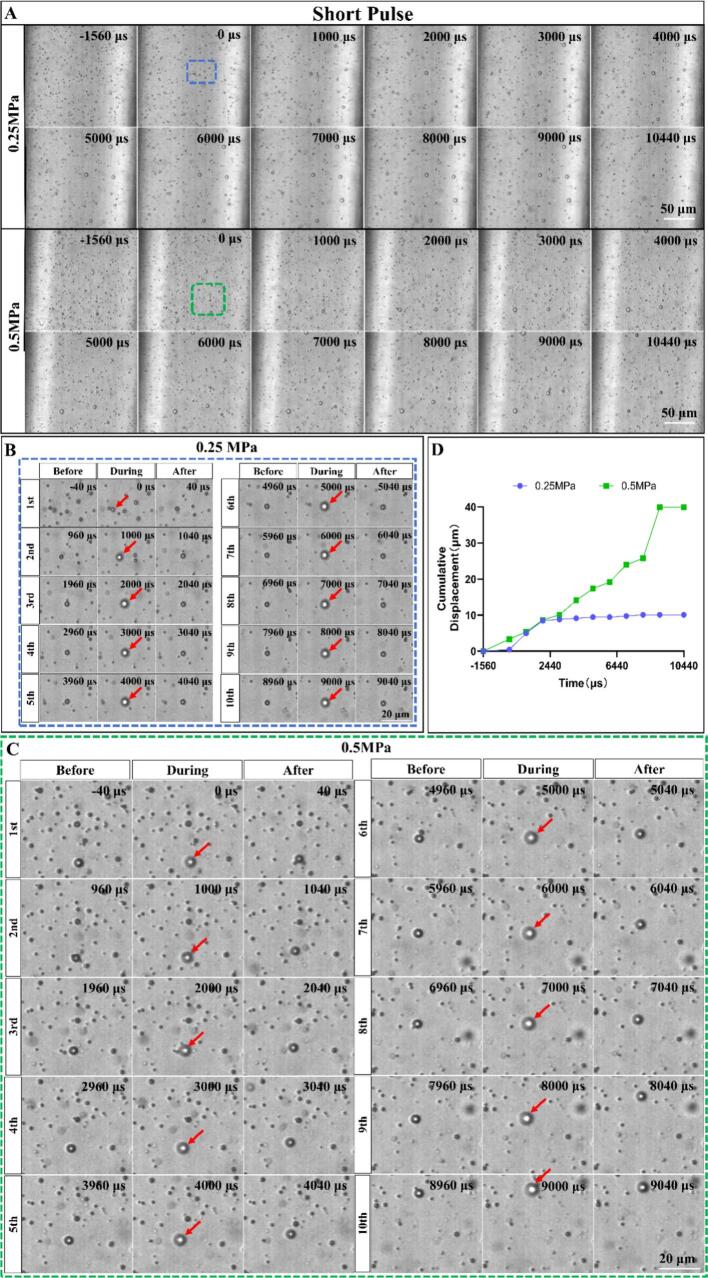
**The dynamic behavior of microbubbles within microchannels under short pulse ultrasound exposure with a flow rate of 75 µL/min.** (A) High speed recordings of bubble population dynamics and spatial distribution under 10 consecutive ultrasound pulse exposure (pulse length 40 µs, pulse interval 1 ms) at 0.25 MPa and 0.5 MPa acoustic pressure. (B-C) The enlarged view of the blue and green dashed box in (A) showing the displacement, volume change and coalescence of individual microbubbles immediately before and after the application of each ultrasound pulse in a 10-pulse train at 0.25 MPa and 0.5 MPa acoustic pressure, respectively. The ultrasound pulse arrives at *t* = 0 and repeats every 1000  μs. (D) The cumulative displacement of the two microbubbles labeled with red arrows in (B) (0.25 MPa) and (C) (0.5 MPa) as a function of time. The exemplary bubble at 0.25 MPa (as shown in B) stopped displacement on the 3rd pulse after it grew to a certain size and then kept oscillating at the same location.

First, we examined the bubble dynamics under short pulse condition by recording 10 consecutive ultrasound pulse exposure (pulse length 40 µs, pulse interval 1 ms). At a lower peak negative pressure of −0.25 MPa, microbubbles exhibited displacement due to acoustic radiation force and mild coalescence. Increasing the acoustic pressure to 0.5 MPa results in more pronounced bubble displacement and coalescence, which led to larger bubbles and a reduced bubble number count (Fig. 3A).

We took a further look into the region enclosed by the blue and green dashed box in Fig. 3A. As pointed to by the red arrows, microbubble expansion occurred with each ultrasound pulse at both 0.25  MPa and 0.5  MPa (Fig. 3 B-C). These two bubbles grew by attracting and coalescence of nearby small bubbles due to the secondary Bjerknes force. Additionally, they are also displaced due to the acoustic radiation force. We find larger translation distances for the higher acoustic pressure of 0.5  MPa (Fig. 3 B-C). Quantitative analysis of the cumulative displacements of the two exemplary bubbles confirmed that the displacement at 0.5 MPa was 4 times that at 0.25 MPa (Fig. 3D).

**Fig. 4:**
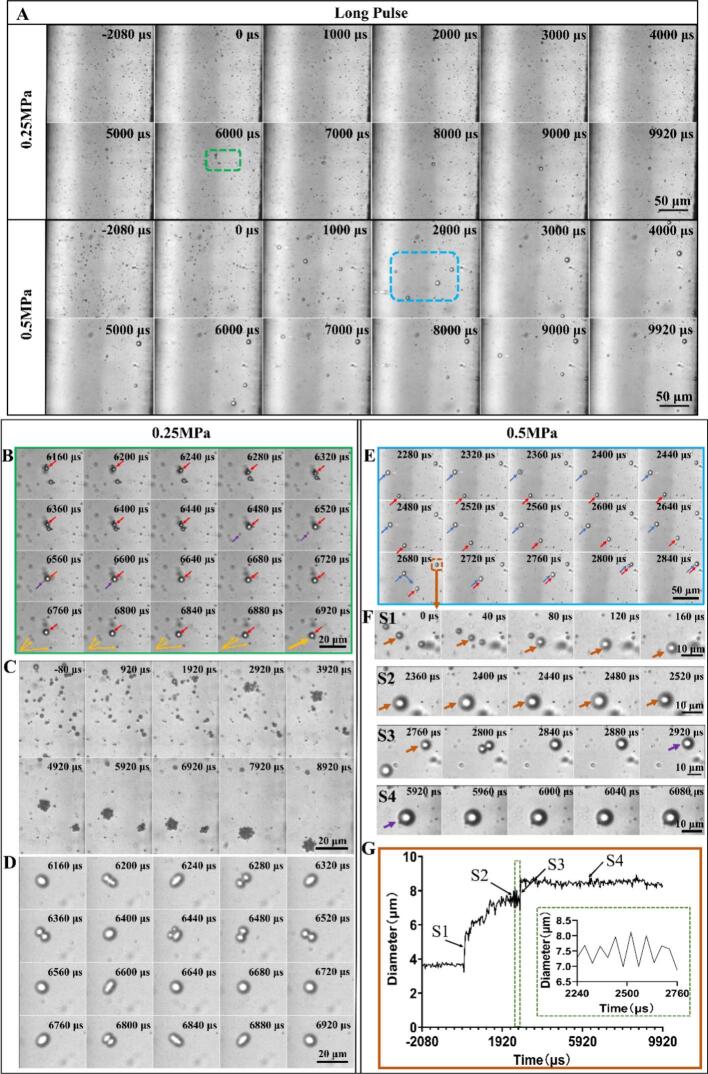
**The dynamic behavior of microbubbles within microchannels under long pulse ultrasound exposure at a flow rate of 75 µL/min.** (A) High speed recordings of the bubble dynamics under one long pulse exposure (pulse length 0 – 9.090 ms) at 0.25 MPa and 0.5 MPa acoustic pressure. Ultrasound begins at *t* = 0. (B) The enlarged view of the bubble dynamics in the green dashed box in (A) showing the displacement and coalescence of microbubbles and (C) a typical example showing the gradual clustering of microbubbles and (D) surface oscillation of an individual microbubble during the long pulse ultrasound exposure at 0.25 MPa. (E) The enlarged view of the bubble dynamics in the blue dashed box in (A) showing the displacement, oscillation and coalescence of microbubbles during the long pulse ultrasound exposure at higher acoustic pressure of 0.50 MPa. (F) Enlarged view of the evolution of the microbubble outlined by brown color (E) at different timings and stages. S1: the microbubble was generated by coalescence of bubble clusters. S2: notable and stable oscillation of the microbubble in the acoustic field. S3: coalescence of the microbubble with another nearby bubble. S4: no discernable oscillation and displacement at this stage even though ultrasound is still on. Please note that S3 has a smaller magnification. (G) Time evolution of the measured bubble diameter corresponding to stage S1-S4 shown in panel F. The inset shows the enlarged view of bubble diameter oscillation in S2.

In the long pulse mode, where each ultrasound pulse lasted 9.09 ms, microbubble displacement, clustering and coalescence were observed (Fig. 4A) (ultrasound is on from 0-9.09 ms). The coalescence resulting in a reduction of the number of bubbles at 0.5  MPa was strongly increased for the long pulses as compared to the short pulses.

We examined in more detail the bubble dynamics at 0.25 MPa and identified three types of bubble behavior. First, as shown in Fig. 4B, an enlarged view of the region enclosed by the green box in Fig. 4A, the microbubble labeled by the red arrow was displaced under ultrasound exposure and subsequently was merged with another microbubble, resulting in a larger bubble size. It further attracted and absorbed smaller surrounding microbubbles (see the purple and orange arrows). Second, we also find in some independent experiments that long pulses triggered bubble clustering rather than coalescence (Fig. 4C). Third, in some circumstances when bubbles coalesced over time to form larger bubbles, they may undergo various surface oscillations (Fig. 4D). This phenomenon is reported to be driven by the Faraday instability [6], [44], which may cause the appearance of half-harmonic patterns, known as shape modes on oscillating bubbles [45], [46], [47], [48].

At higher acoustic pressure of 0.5 MPa in the long pulse mode, bubbles showed displacement and coalescence already during the first 2  ms as compared to 0.25 MPa amplitude, where coalescence and reduction of the bubble number started only from 3 ms on. We further examined selected individual bubble dynamics in the dashed blue box in Fig. 4A. There the microbubble tracked by the blue arrow was first displaced and coalesced with the one tracked by a red arrow (Fig. 4E) and finally merged with the microbubble in the upper right corner (in brown dashed box) during *t* = 2280–2840 μs. We then took a closer look of the time evolution of the microbubble in the brown box (labeled by the brown arrow in Fig. 4F). It was formed through the coalescence of nearby bubbles during the first 160 μs of ultrasound exposure (stage S1) and then underwent periodic oscillation during the interval 2360 μs < *t* < 2520 μs (stage S2). Later it merged with another large microbubble nearby increasing its size further (stage S3) and then demonstrated no discernable oscillation till the end of the ultrasound pulse (stage S4). This may be due to the shift of its resonance frequency. The temporal evolution of the volume-weighted average diameter of the microbubble labeled in brown throughout the ultrasound pulse exposure and its enlarged oscillation curve in stage S2 is shown in Fig. 4G. It revealed the rapid growth of the bubble in stage S1 through coalescence up to stage S2 (*t* < 2240 μs), a stable oscillation in stage S2 and further coalescence and growth in stage S3 before it became almost quiescent in stage S4.

**Fig. 5:**
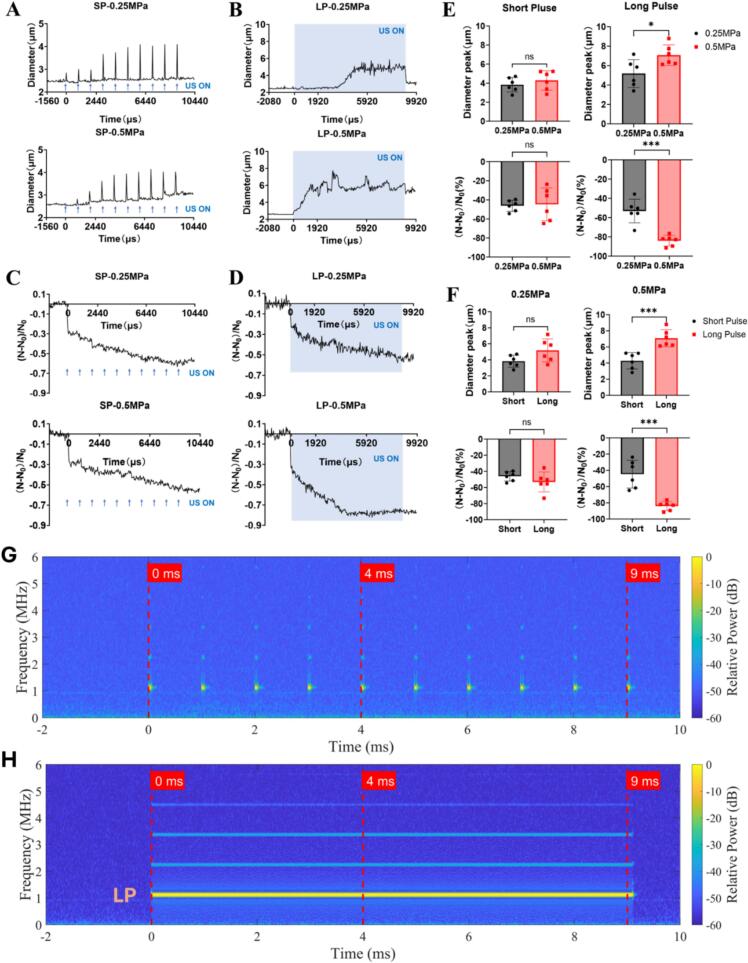
**Analysis and quantification of bubble size and number under short and long pulse ultrasound exposure.** (A) The time evolution of the volume-weighted diameter of microbubbles under 10 consecutive short pulses (SP) for 0.25  MPa and 0.50 MPa acoustic pressure. The timing of each ultrasound pulse is indicated by blue arrows. (B) The time evolution of the volume-weighted diameter of microbubbles under one long pulse at 0.25  MPa and 0.50  MPa acoustic pressure. Ultrasound exposure is indicated with the shaded areas. (C) The relative bubble number change (N – N_0_) / N_0_ for 10 consecutive short pulses at 0.25  MPa and 0.50 MPa acoustic pressure. (D) The relative bubble number change (N – N_0_) / N_0_ for one long pulse at 0.25  MPa and 0.50 MPa acoustic pressure. (E) Statistical analysis and comparison of the relative bubble number change and the peak value of the volume-weighted diameter between 0.25 MPa and 0.5 MPa for the short pulse and long pulse mode. (F) Statistical analysis and comparison of the relative bubble number change and the peak value of the volume-weighted diameter between short pulse and long pulse mode at 0.25  MPa or 0.5  MPa. Student *t*-test is used, N = 6 independent chip experiments for each group: *p < 0.05, ***p < 0.001. Passive cavitation detection (PCD) measurement and spectra analysis for short pulse (G) and long pulse (H) at an acoustic pressure of 0.5 MPa.

Next, we calculated the volume-weighted diameter of the bubbles in each image frame and their evolution over time (Fig. 5A and Fig. 5B) to assess bubble expansion and coalescence. We further counted the total number of microbubbles in each image frame and their change ($\frac{N-N_{0}}{N_{0}})$ over time (Fig. 5C and Fig. 5D) for quantification of bubble coalescence or destruction.

As shown in Fig. 5A, under short pulse mode, the average diameter of the microbubbles increased and peaked during each brief ultrasound exposure (40 µs) 10 times, which is due to negative acoustic pressure driven bubble volume expansion. These peaks showed an upward trend with the number of ultrasound pulse exposure. The average bubble diameter returned back to about its initial value after each short ultrasound pulse at 0.25 MPa while it showed an accumulative effect on the baseline bubble diameter at 0.5 MPa. This may be attributed to more profound bubble coalescence at 0.5 MPa. Under long-pulse mode, the averaged bubble diameter kept growing during the 9.09 ms–long ultrasound exposure at 0.25 MPa while it first increased sharply upon the onset of the ultrasound then varies around a high-level baseline at 0.5 MPa. There is no statistical difference in the peak value of the average bubble diameter between 0.25 and 0.50 MPa under short pulse exposure, but significant difference between 0.25 and 0.50 MPa was measured for the long pulses (Fig. 5E).

For the bubble number change under short pulse, both 0.25 MPa and 0.50 MPa demonstrated an initial sharp reduction for the 1st short pulse followed by a more gradual decrease for the later pulses (Fig. 5C). For the long pulses, the bubble number showed a similar reduction at the onset of the ultrasound and a slower decay at the later stages (Fig. 5D). There is no statistical difference in the relative bubble number change between 0.25 MPa and 0.50 MPa for the short pulse exposure, but significant difference between 0.25 MPa and 0.50 MPa was measured for the long pulse (Fig. 5E).

We further compared the difference for the change of bubble diameter and bubble number between short and long pulses. There is no statistical difference in both the bubble diameter peak and the relative bubble number change at 0.25 MPa. In contrast, significant difference (p < 0.001) in both the bubble diameter peak and the relative bubble number change was measured at 0.50 MPa between short and long pulses (Fig. 5F). This difference likely arose from the more sustained acoustic effects for the long pulse mode, where prolonged ultrasound exposure intensifies cavitation activity, leading to stronger microbubble coalescence and bubble size growth.

We further performed passive cavitation detection (PCD) measurement to define the exact cavitation regime. As shown in Fig. S4, the water control (without microbubbles) at acoustic pressure of 0.5 MPa exhibits the spectrum of the fundamental frequency ($f_{0}$) only and a flat baseline. In contrast, under both the short pulse (SP) and long pulse (LP) conditions (Fig. 5G and Fig. 5H), the microbubble groups exhibit higher harmonic emissions (e.g., $2f_{0}$, $3f_{0}$). This provides evidence that microbubbles are undergoing stable cavitation and our system is operating below the inertial cavitation threshold. For more details, please refer to the supporting information, Fig. S4 and Fig. S5.

Furthermore, since the SP condition involves intermittent sonication, it is critical to confirm that microbubbles are not rapidly destroyed after the initial acoustic cycles. As detailed in the zoomed-in panels of Fig. S4(b) (capturing individual bursts at 0 ms, 4 ms, and 9 ms), the microbubbles consistently exhibit identical stable cavitation spectra without any stochastic broadband noise that would indicate inertial collapses. This confirms the pulse-to-pulse consistency of the stable cavitation regime across the entire stimulation window. Similar stable cavitation signatures were also found at the lower acoustic pressure of 0.25 MPa (Fig. S6 and Fig. S7).

### 3.3 Cellular bioeffects induced by short pulse and long pulse ultrasound exposure

Ca^2+^ signaling has been reported to influence cell fate after sonoporation and regulate tight junction opening [4], [7], [23]. Therefore, we performed concurrent Ca^2+^ imaging and PI imaging (indicating membrane permeability) for bEnd.3 monolayer culture in the vessel-mimicking channels under short pulse and long pulse ultrasound stimulation.

**Fig. 6:**
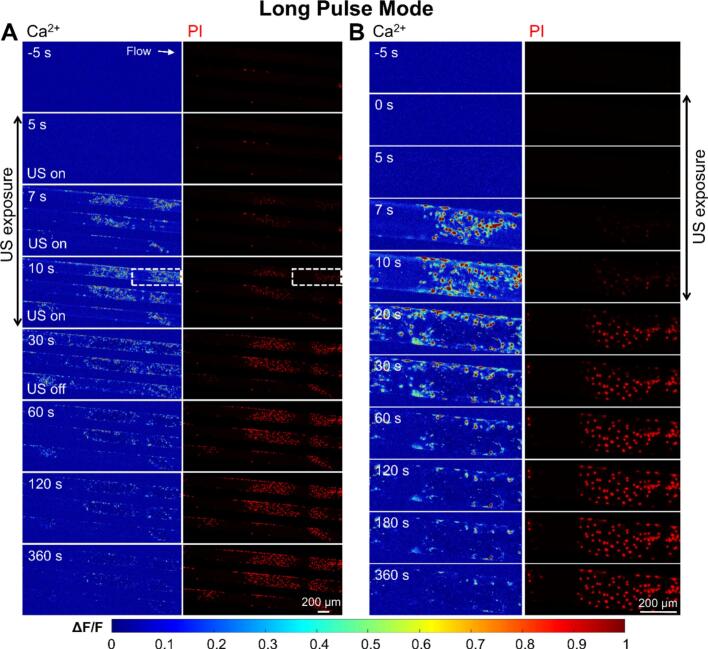
**Characteristics of cellular Ca^2+^ signaling and membrane poration of bEnd.3 monolayer culture in the vessel-mimicking channel under 0.5 MPa long pulse ultrasound exposure for 10 s at a flow rate of 75 ul/min.** (A) Wide-field imaging of cellular Ca^2+^ dynamics (Fluo-4, pseudo color) and PI uptake (red) in a microfluidic chip before, during (0 < *t* < 10 s), and after the long pulse ultrasound stimulation (*t* > 10  s). The left column indicates the normalized Ca^2+^ change (ΔF/F_0_) with warmer colors representing a stronger Ca^2+^ response. The right column displays PI fluorescence, highlighting membrane-compromised cells. (B) Enlarged view of the selected region from Panel A, showing the progression of cellular Ca^2+^ signaling and membrane poration at higher resolution. The same time labels and scale bars are shared between the columns in each panel.

Under long pulse stimulation, cells exhibited topical and intense Ca^2+^ response after 7 s from ultrasound initiation (t = 0) (Fig. 6A), which further expanded to neighboring region at *t* = 10 s. Later, cells showed gradually PI uptake until reaching a plateau while Ca^2+^ signaling declined to baseline level. It is worth noting that in the regions with notable PI uptake, the cellular Ca^2+^ peaked between 7–10 s but became significantly lower later, likely due to fluo-4 dye leakage from excessive membrane poration (see the enlarged view at t = 20–30 s in Fig. 6B). No membrane detachment was observed.

**Fig. 7:**
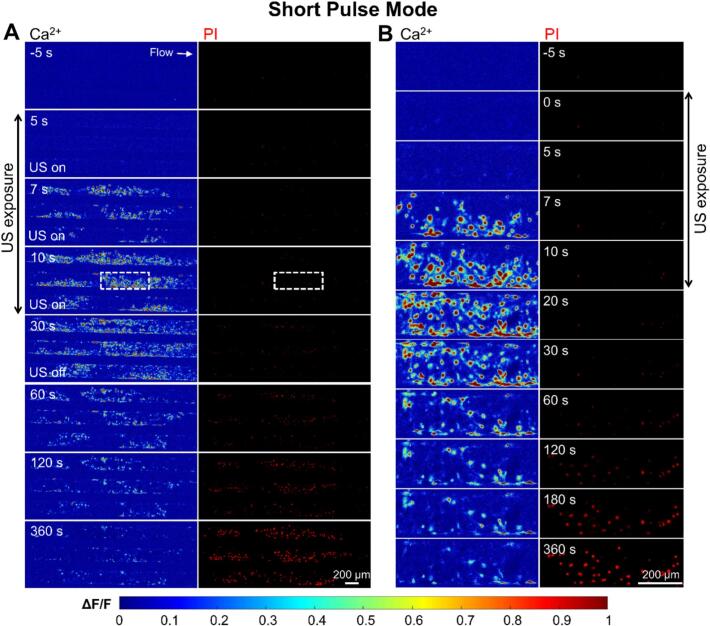
**Characteristics of cellular Ca^2+^ signaling and membrane poration of bEnd.3 monolayer culture in the vessel-mimicking channel under 0.5 MPa short pulse ultrasound exposure for 10 s at a flow rate of 75 ul/min.** (A) Image sequences showing Ca^2+^ signalling (Fluo-4, pseudo color, left column) and PI uptake (red, right column) before, during (0–10 s), and after ultrasound exposure at short pulse mode. (B) Enlarged view of the dashed box in (A) showing the time evolution of Ca^2+^ signalling (left) and PI uptake (right). The two channels share the same time labels and scale bar. Ultrasound is on from 0-10 s.

In contrast, the 10 s exposure of short pulse ultrasound elicited more uniform Ca^2+^ response across the cell population and milder membrane poration (Fig. 7A). Interestingly, the Ca^2+^ response lasts longer at a high level (from 7 s < t < 80 s) as compared to the long pulse conditions (see the enlarged view in Fig. 7 B). The PI uptake also became slower and weaker. These demonstrated that short pulses promoted Ca^2+^ signaling without causing excessive membrane poration or damage.

**Fig. 8:**
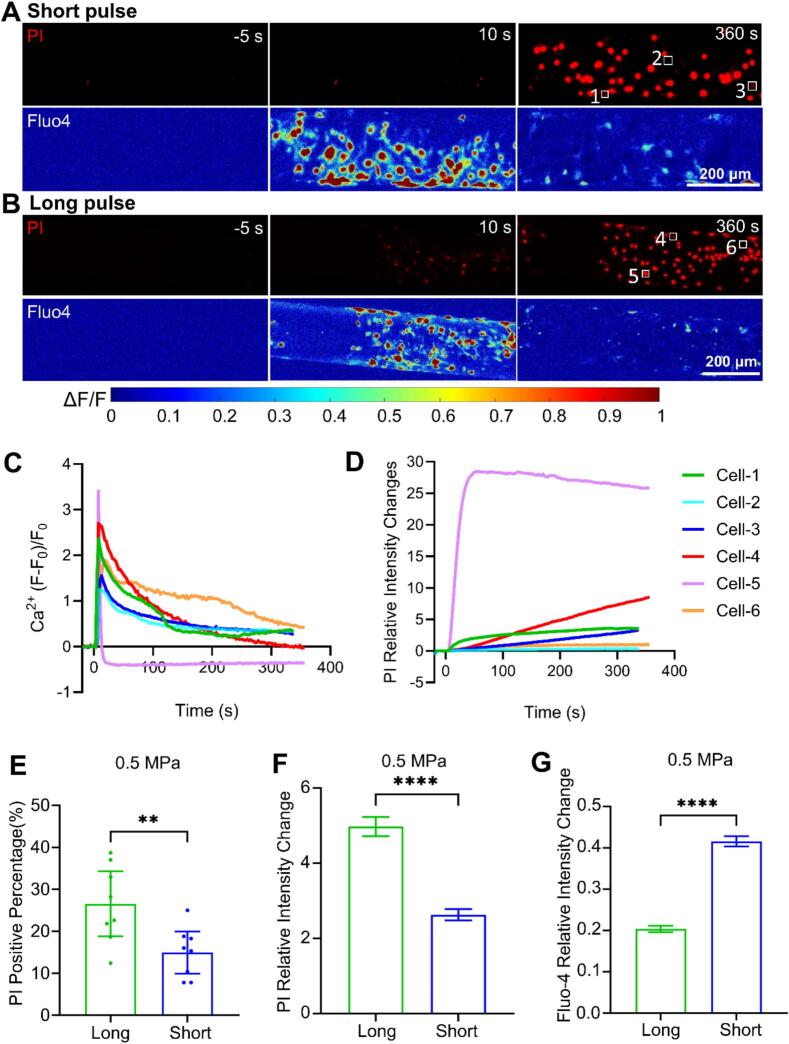
**Comparison of generated cellular Ca^2+^ signaling and membrane poration between short pulse and long pulse treatment for 10 s at 0.5 MPa acoustic pressure. (A)** and **(B)** PI uptake (red, upper panel) and Ca^2+^ signaling (pseudo color, bottom panel) in bEnd.3 cell monolayer in the microchannels before (−5 s), during (10 s), and after (360 s) 0.5 MPa ultrasound exposure at long pulse and short pulse mode, respectively. **(C)** Ca^2+^ response ΔF/F vs. time for exemplary cells labeled 1 to 3 at short pulse mode (A) and 4 to 6 at long pulse mode in (B). **(D)** Relative PI uptake (I-I_0_)/I_0_)_PI_ vs. time for the cells labeled 1 to 3 in the short pulse mode and 4 to 6 in the long pulse mode. The same color coding for cells is used in panel C and D. **(E)** The percentage of cells showing PI uptake in each independent microfluidic chip experiment. *n* = 8 chips for each group. **(F)** and **(G)** PI relative intensity change and Ca^2+^ response peak values from individual cells in the above experiments. In panel F, N = 11047 and 11,100 cells for long and short pulse mode, respectively. In panel G, N = 11290 and 11,300 cells for long and short pulse, respectively. The student *t*-test was used for statistical analysis. **p < 0.01, ***p < 0.001, ****p < 0.0001. Please note that in panel E the statistical tests treat chip-level replication as the experimental unit while panel F and G treat individual cells as independent units.

We further examined individual cells treated by the two pulse sequences at 0.5 MPa acoustic pressure (Fig. 8). Representative cells labeled by 1 to 3 in the short pulse mode (Fig. 8A) and 4 to 6 (Fig. 8B) in the long pulse mode demonstrated differential PI uptake and Ca^2+^ signaling following ultrasound exposure. Cell #2 and #6 exhibited no discernable membrane poration but mild Ca^2+^ signaling that reached a lower peak value than the other perforated cells (Fig. 8C and Fig. 8D). Interestingly, although the final PI uptake in cell #4 and 5 (long pulse) was more than 2 times larger than that in cell #1 and 3 (short pulse) (Fig. 8D), the peak values of Ca^2+^ signaling of the latter were similar to those of the former cells (Fig. 8C). The results also demonstrated that cell #5 experienced excess membrane poration and Ca^2+^ indicator leakage with long pulse treatment. We further conducted quantitative analysis to compare the variations in PI uptake and Ca^2+^ signaling across each independent experiment under the two pulse sequences. The percentage of PI-positive cells and the relative PI intensity change were both significantly higher under long pulse mode (Fig. 8E and F). However, the magnitude of Ca^2+^ response was substantially higher in the short pulse mode compared to the long pulse mode (Fig. 8G). These findings reveal that pulse duration is a key determinant in ultrasound-mediated cellular responses.

### 3.4 Mechanistic study of cellular calcium signaling induced by short pulse ultrasound

**Fig. 9:**
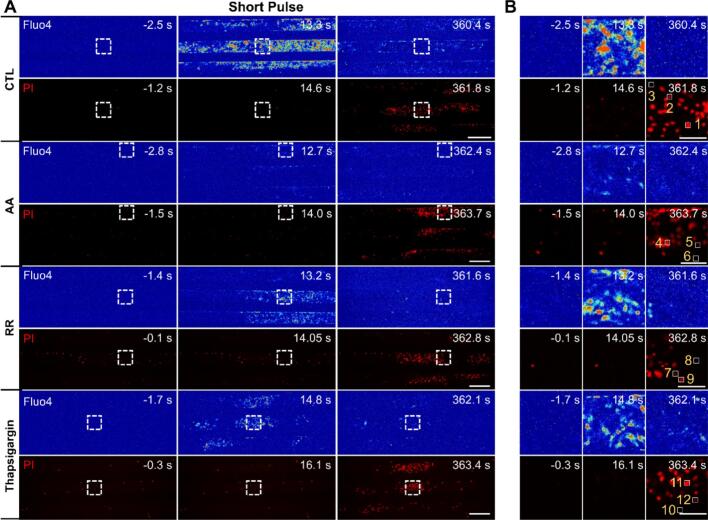
**Mechanistic study for cellular Ca^2+^ response induced by 10 s short pulse ultrasound stimulation at 0.5 MPa acoustic pressure at a flow rate of 75 ul/min. (A)** Image sequences of induced Ca^2+^ signaling (Fluo-4, pseudocolor) and PI uptake (red) for control groups without inhibitors, treated by ascorbic acid (AA), Ruthenium Red (RR) and Thapsigargin. Ultrasound is on from 0 to 10 s. The scale bars denote 400 µm. **(B)** Enlarged views of the dashed boxes in panel (A) in corresponding rows. The scale bars denote 100 µm.

Considering the efficiency and biosafety of evoking cellular Ca^2+^ response, we performed mechanistic study under 10 s short pulse stimulation at 0.5 MPa acoustic pressure. Without any inhibitors (CTL), cell monolayers showed obvious Ca^2+^ response soon after ultrasound exposure (Fig. 9A and B). Cavitation activity is known to generate reactive oxygen species (ROS) that may affect cellular bioeffects. After introducing ascorbic acid (AA) to scavenge ROS, ultrasound induced Ca^2+^ response significantly reduced although the cells showed membrane poration to a similar extent. When inhibiting mechanosensitive cation channels with ruthenium red (RR), cellular Ca^2+^ signaling moderately decreased compared to control group. Similarly, when cells were treated with thapsigargin to deplete internal Ca^2+^ storage, cellular Ca^2+^ signaling reduced noticeably but not drastically. With reduced Ca^2+^ responses in these conditions, PI uptake and cell perforations were still observed. We selected three exemplary cells for further analysis from each condition, as labeled by #1 to #12 in Fig. 9B.

**Fig. 10:**
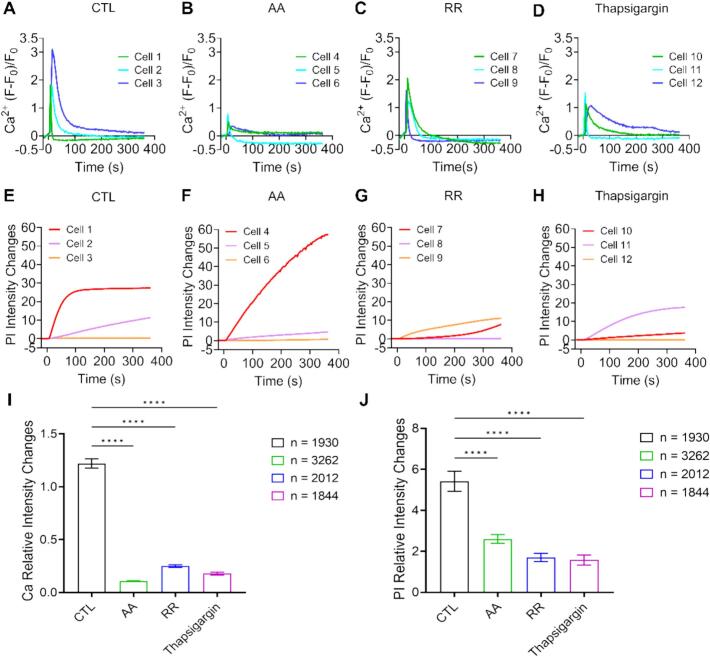
**Exemplary cells and statistical analysis for the mechanisms of cellular Ca^2+^ response induced by 10 s short pulse ultrasound at 0.5 MPa.** Time traces of Ca^2+^ response ΔF/F for exemplary cells labeled by 1 to 3 in the control group (A), 4 to 6 in the AA group (B), 7 to 9 in the RR group (C) and 10 to 12 in the Thapsigangin group (D). (E-H) The relative PI uptake vs. time for the above exemplary cells. (I) and (J) Statistical analysis of the Ca^2+^ signaling and PI uptake from all individual cells between different groups. Significant differences were determined by One-Way ANOVA on all individual cells; ****p < 0.0001. The total number of cells in each group is indicated in the figure legend.

Compared to cells 1 to 3 from the control group (Fig. 10A), cells 4 to 6 showed notable suppression of Ca^2+^ responses with AA treatment to eliminate ROS (Fig. 10B). Cells 7 to 8 from RR treatment blocking mechanosensitive ion channels and cells 10 to 12 with Thapsigargin affecting internal release demonstrated lower peaks of Ca^2+^ transients (Fig. 10 C-D). The PI uptake of cells 5 to 12 exhibited a moderate decrease compared to the control group (Fig. 10 E-H). Statistical anlaysis of all the cells from each group confirmed that AA treatment affected cellular Ca^2+^ response to the largest extent (Fig. 10I). This finding strongly suggests a critical role for ROS in mediating ultrasound-induced Ca^2+^ signaling. All these inhibitors could significantly reduce cellular Ca^2+^ transients and PI uptake (Fig. 10 I–J), suggesting that ROS, mechanosensitive ion channels and internal Ca^2+^ release contributed to ultrasound induced Ca^2+^ signaling and membrane poration.

### 3.5 Molecular transport across endothelial layer induced by long and short pulse ultrasound

Next, we further investigated the molecular transport across the endothelial monolayer generated by long and short pulse ultrasound with the transwell model. Both membrane poration and Ca^2+^ signaling may contribute to the molecular transport. We evaluated the effect of the total treatment time and pulse length on the transport of molecules of different size, i.e., 10 kDa and 40 kDa FITC-dextran.

**Fig. 11:**
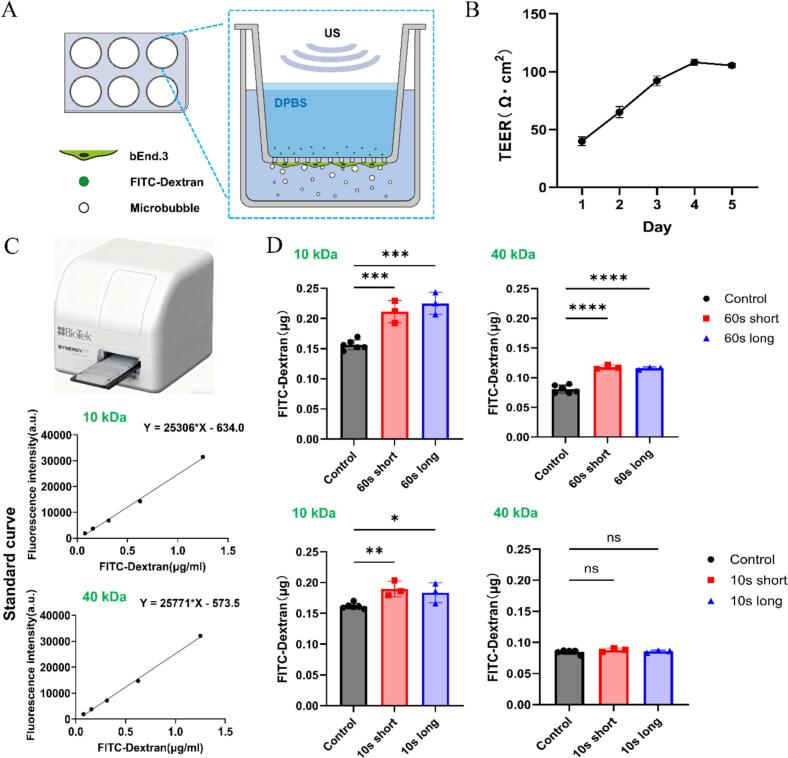
Trans-endothelial transport of 10 kDa and 40 kDa FITC-dextran with ultrasound treatment at short and long pulses. (A) Schematic of the experimental apparatus in transwell. (B) Measured *trans*-endothelial electrical resistance (TEER) in the transwell from Day 1 to Day 5 after cell seeding. (C) Schematic of the microplate reader (upper) and the standard curves for 10  kDa and 40  kDa FITC-dextran measured by the microplate reader (bottom). (D) Quantification of the total amount of transported 10  kDa and 40  kDa FITC-dextran to the bottom well at 1 h after ultrasound stimulation in different scenarios with microplate reader. 60 s or 10 s short pulse and long pulse were used here. Significant differences were determined by One-Way ANOVA, *n* = 3–6 wells for each group, *p < 0.05, **p < 0.01, ***p < 0.001, ****p < 0.0001.

To ensure thorough contact between microbubbles and cells, we cultured the bEnd.3 cells at the bottom of the transwell insert to form a monolayer and added microbubbles to the bottom chamber so that they can rise to the cell surface due to buoyancy (Fig. 11A). Experiments were only performed when the endothelial layer reached its peak integrity (i.e., TEER value above 100 ῼ∙cm^2^) (Fig. 11B). The microplate reader was utilized to measure the amount of FITC-dextran transported from the upper chamber to the bottom of the transwell by calibration with standard curves, which convert fluorescent intensity to molecule concentrations (Fig. 11C). Statistical results show that 60  s of short pulse and long pulse ultrasound duration both significantly increased the total amount of 10 kDa and 40 kDa dextran transport across the endothelial layer compared to the control group. Yet the delivered amount was higher for the 10 kDa dextran. However, under 10 s ultrasound exposure, neither short pulse nor long pulse could achieve significant enhancement for the delivery of 40 kDa dextran while they still significantly enhanced the transport of 10 kDa dextran, though slightly less compared to that under 60 s ultrasound exposure. No significant difference was observed between short and long pulses. These results indicate that the total ultrasound treatment time plays an important role for molecular transport. Moreover, both membrane poration and Ca^2+^ signaling, and their tradeoff, determins the final molecular transport.

## 4 Discussion

### 4.1 Pulse length dependent interplay between bubble dynamics and cellular bioeffects

The therapeutic promise of ultrasound-mediated drug delivery is constrained by a fundamental challenge: how to achieve sufficient barrier permeabilization while maintaining cellular viability and function [2]. Although cavitation activity is widely recognized as the primary physical mechanisms of sonoporation, the downstream biochemical signaling events that ultimately dictate cellular fate, e.g., whether cells recover or die, remain insufficiently understood. This study provides a mechanistic investigation into how acoustic parameters of ultrasound pulses differentially regulates microbubble dynamics, calcium signaling, and endothelial barrier permeability. By employing a vessel-mimicking microfluidic platform that integrates real-time, high-resolution observation of bubble dynamics, live-cell Ca^2+^ responses, and membrane perforation, we propose that ultrasound pulse structure is a critical parameter that shapes not only bubble behavior but also the spatiotemporal profile and physiological consequences of Ca^2+^ signaling, a key regulator of endothelial integrity.

The response of microbubbles under ultrasound exposure is crucial for understanding the mechanical forces exerted on cells. Our high-speed imaging results demonstrate that microbubble displacement, clustering and coalescence were significantly influenced by ultrasound pulse duration and acoustic pressure. Under SP mode at 0.5 MPa, bubble expansion is transient and reversible, with limited coalescence and moderate displacement. In contrast, LP ultrasound at 0.5 MPa led to significantly more pronounced bubble clustering, coalescence, and prolonged oscillation. Therefore, microbubble distribution was more uniform following short-pulse exposure, as reported by previous studies [32], [35], in contrast to the markedly diminished bubble population under long-pulse exposure. These differences in bubble dynamics and spatiotemporal distribution directly translate into varied mechanical stimuli delivered to the endothelial monolayer. The increased microbubble coalescence and prolonged oscillation under LP mode likely contribute to the higher degree of membrane permeabilization observed in our experiments. This is supported by the higher percentage of sonoporated cells and stronger PI fluorescence intensity under LP compared to SP condition. It is worth noting that the translational microbubble dynamics observed in our vessel-mimicking microchannels, including displacement, clustering, and coalescence, are not in vitro artifacts but have been reported in vivo [49], [50], validating the physiological relevance of our observations. Although we did capture some coarse bubble oscillation information (e.g., Fig. 4D, F, G), resolving more detailed radial oscillations would require higher frame rates that is beyond the capability of our Photron camera.

A key finding of this work is the identification of distinct, pulse-length-dependent Ca^2+^ signaling profiles. A 10  s SP ultrasound stimulation induces a more uniform and wider spreading Ca^2+^ elevation across the cell population as compared to a 10  s LP that avoided cell detachment. This response was associated with mild cell membrane poration or no sonoporation. Ca^2+^ signaling can be initiated either by extracellular Ca^2+^ influx through sonoporation pores or mechanosensitive channels, or by the release from intracellular stores like the endoplasmic reticulum (ER) [4], [51]. This initial signal often evolves into an intracellular Ca^2+^ wave, which can propagate via gap junctions to neighboring cells, triggering intercellular waves [4], [52]. These Ca^2+^ signaling characteristics are consistent with the uniform microbubble distribution and resultant mechanical stimulation from SP ultrasound. Importantly, we observe that Ca^2+^ signaling peaks was significantly higher at SP conditions despite higher percentage and larger extent of sonoporation found at LP exposure. One possible reason could be due to Ca^2+^ indicator leakage from the more intense membrane poration at LP conditions. Another reason could be a more efficient and global activation of Ca^2+^ signaling pathways under SP mode.

Through pharmacological inhibition, we dissected the contributions of key pathways to ultrasound-induced Ca^2+^ signaling at SP conditions. ROS emerged as a major mediator, as scavenging with ascorbic acid substantially blunted Ca^2+^ responses. It is known that microbubble-assisted ultrasound could induce ROS production[53], [54], [55]. In our experiment, ROS is generated in stable cavitation regime, which is consistent with previous study [55]. Cavitation-generated ROS may act on redox-sensitive ion channels (e.g., transient receptor potential (TRP) channels in the plasma membrane), or modulate Ca^2+^ release channels (e.g., IP_3_ and ryanodine receptors) on the sarcoplasmic/endoplasmic reticulum (SR or ER) membrane, to initiate and amplify Ca^2+^ signaling [56], [57]. Further inhibition of mechanosensitive channels with ruthenium red or depletion of intracellular Ca^2+^ stores with Thapsigargin each reduced but did not abolish Ca^2+^ transients, indicating that multiple pathways operate in concert. These findings highlight the multimodal nature of ultrasound-triggered Ca^2+^ signaling, involving both physical (mechanical) and biochemical (ROS) components. It indicates ROS not merely work as cytotoxic byproducts of inertial cavitation but also as potent signaling molecules at mild dose, capable of modulating endothelial function through redox-sensitive pathways.

### 4.2 Implications for endothelial barrier modulation and ultrasound-mediated therapeutic delivery

The differential cellular responses to SP and LP ultrasound, where SP favored widespread Ca^2+^ signaling and LP induced focal sonoporation, highlight a critical balance between signaling-mediated and poration-mediated transport. This balance offers a plausible explanation for the comparable molecular delivery efficiencies observed for both pulse modes in our transwell experiments. For the 10 s exposure (which avoided cell detachment), both modes achieved similar 10 kDa dextran delivery. This outcome is consistent with a model where enhanced paracellular transport from robust Ca^2+^ signaling under SP compensates for its reduced direct transcellular poration, while under LP exposure, efficient transcellular poration compensates for its more heterogeneous Ca^2+^ signaling. At the longer 60 s treatment that improved 40 kDa dextran delivery, a similar principle may apply: each pulse mode leverages its dominant mechanism, SP through sustained Ca^2+^-mediated tight junction modulation and LP through higher percentage and larger extent of membrane poration or some topical spots of cell detachment, to achieve a comparable net increase in barrier permeability for larger molecules. This suggests that the total therapeutic effect of ultrasound on molecular transport is not dictated by a single mechanism but may be an integrated output of both paracellular (Ca^2+^-driven) and transcellular (sonoporation-driven) pathways.

However, we acknowledge that the direct attribution of these transport pathways remains speculative without specific structural or functional evidence. While our data demonstrate wider Ca^2+^ wave propagation under SP and higher sonoporation rates under LP, we did not perform post-ultrasound measurements of *trans*-endothelial electrical resistance (TEER) or visualize tight junction (TJ) remodeling. Such experiments would be required to definitively confirm a shift in the paracellular route. Therefore, our interpretation of paracellular vs. transcellular transport is presented as a mechanistic hypothesis derived from the observed patterns of Ca^2+^ signaling, membrane poration, and molecular flux, rather than a definitive conclusion. Future work could directly assess junctional integrity and function to validate this proposed mechanism.

It is worth noting that the above transwell molecular transport results were confined to 10 s and 60 s total ultrasound exposure and may not apply to even longer treatment (e.g., 120 s). Further extending total treatment time can boost delivery efficiency. However, this gain may be counterbalanced by the propensity for excessive damage and cell detachment, especially under long pulses, which would disrupt the equilibrium of paracellular and transcellular transport mechanisms. It is also worth noting that there could be potential bias introduced by dye leakage. Despite this, several lines of evidence support our interpretation that short pulse (SP) ultrasound preferentially engages paracellular transport pathways: (i) the wider spatial propagation of intercellular Ca^2+^ waves observed under SP conditions, (ii) the significantly lower rates of direct sonoporation at the endothelial barrier compared to long pulse (LP) exposures, and (iii) the comparable overall molecular transport across the endothelial monolayer between SP and LP conditions in Transwell assays. Taken together, these findings indicate that paracellular transport may contribute substantially to the enhanced permeability observed with SP ultrasound, compensating for its lower direct sonoporation efficiency.

In this study, we established a compact, acoustically coupled, vessel-mimicking microfluidic system under physiological flow to resolve microscopic mechanisms. The custom-built ring transducer, precisely aligned with the microchannels, enables simultaneous sonication and high-resolution optical imaging, providing a powerful platform for future studies. Our simplified microfluidic model, which, while offering good control of bubble-cell interactions and real-time imaging, does not fully recapitulate the complexity of in vivo vasculature, including shear stress adaptation and interactions between endothelial cells and other cell types [23], [58]. Another limitation of this study is that we used homemade bubbles and murine endothelial cells. More clinically relevant bubbles and human cells are warranted to be used in future studies. Nevertheless, current findings may still hold as our homemade bubbles exhibit size distribution characteristics comparable to Definity, and similar mechanobiological Ca^2+^ signaling increased paracellular permeability for human cerebral endothelial cell/D3 (hCMEC/D3) monolayer [59]. It should be noted that the fibronectin coating used to promote cell adhesion under flow in our study may itself influence endothelial signaling, as different matrices can modulate mechanotransduction and calcium homeostasis under flow [60]. While our consistent coating allows direct comparison between pulse modes, future work may explore whether alternative matrices alter the cellular response to ultrasound, and explore how pulse duration influences Ca^2+^ signaling in more physiologically relevant models, such as co-culture systems. Additionally, the molecular identity of the ROS-sensitive Ca^2+^ pathways warrants further investigation. In current study, ROS involvement is inferred pharmacologically, and direct ROS measurements could be performed in future work.

## 5 Conclusion

This study establishes ultrasound pulse length and total treatment time as critical modulators of endothelial Ca^2+^ signaling and barrier permeability. Long pulses (10 s) generated more extensive membrane poration, whereas short pulses elicited wider, more robust Ca^2+^ signaling. This Ca^2+^ response is mediated by ROS, mechanosensitive ion channels, and internal store release. Both pulse modes enhanced 10 kDa dextran transport, while extended treatment (60 s) improved 40 kDa delivery. Based on these integrated observations of bubble dynamics, Ca^2+^ signaling, sonoporation, and molecular transport, we propose that acoustic parameters can be tuned to balance Ca^2+^-mediated paracellular transport with sonoporation-driven transcellular delivery. While direct validation of these specific transport routes (e.g., via tight junction staining) is warranted in future studies, this work provides a foundational framework linking pulse-dependent bubble dynamics to distinct cellular signaling mechanisms. These findings contribute to the rationale for designing targeted ultrasound protocols, from direct, intensive drug/gene delivery (favoring long pulses) to transient, potentially safer barrier opening (favoring short pulses).

## 6 Data availability

All data supporting the findings of this study are available within the article and its supplementary files. Any additional requests for information can be directed to and will be fulfilled by the corresponding authors.

## CRediT authorship contribution statement

**Chaofeng Qiao:** Writing – original draft, Methodology, Investigation, Formal analysis. **Siyu Luo:** Writing – original draft, Visualization, Investigation, Formal analysis. **Zhihui Liu:** Writing – original draft, Visualization, Investigation, Formal analysis. **Yingxuan Bu:** . **Yicong Cai:** Methodology, Investigation. **Zhuoyan Liu:** Methodology, Investigation. **Liying Wang:** Writing – review & editing, Writing – original draft, Visualization, Supervision, Methodology, Formal analysis. **Claus Dieter Ohl:** Writing – review & editing, Writing – original draft, Visualization, Supervision, Software, Methodology, Funding acquisition, Formal analysis, Conceptualization. **Fenfang Li:** Writing – review & editing, Writing – original draft, Visualization, Supervision, Software, Methodology, Funding acquisition, Formal analysis, Conceptualization.

## Declaration of competing interest

The authors declare that they have no known competing financial interests or personal relationships that could have appeared to influence the work reported in this paper.
