# Supplementary material for: Tuning endothelial barrier permeability with ultrasound: a pulse-length-dependent interplay between bubble dynamics and cellular bioeffects
Source: Ultrason Sonochem. 2026 Apr 12;129:107851. doi: 10.1016/j.ultsonch.2026.107851 (PMC13139967; doi:10.1016/j.ultsonch.2026.107851)
Supplement: Supplementary Data 1 [file mmc1.pdf]

Supplementary Materials

This file includes:

Supplementary Text

Figs. S1 to S7

Movies S1 to S4

Supplementary Text

1. Acoustic Transmission Measurement

To prevent macroscopic standing waves and bottom reflections during ultrasound exposure, we custom-designed a 3D-printed acoustic absorber array (featuring a pyramid-structured completely solid layer) placed at the bottom of the water tank. Additionally, the vessel-mimicking phantom was mounted on an open-window floating stage, ensuring a completely unblocked acoustic path beneath the focal region (**Figure S1**).

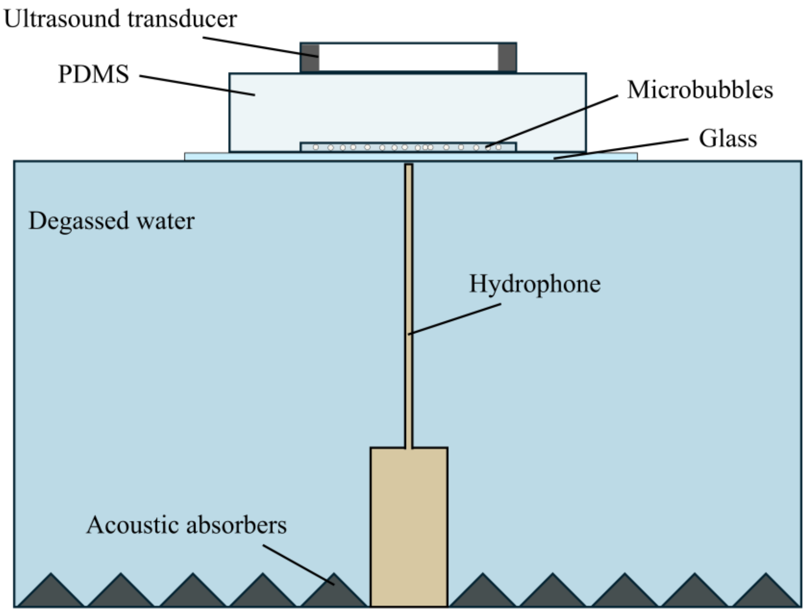

Figure S1. Schematics of the experimental setup for acoustic measurement.

We measured the acoustic transmission through the PDMS channel with and without the glass substrate using a calibrated needle hydrophone (SN: 4536, Precision Acoustics Ltd., UK). As shown in **Figure S2**, a steady-state window (from 12 μs to 28 μs) was extracted for analysis to ensure accuracy. The effective acoustic pressure amplitude was represented by the Root Mean Square (RMS) voltage within this steady-state region[1]. The measured RMS values were 8.49 mV (without glass) and 7.36 mV (with glass). The acoustic Transmission Loss (TL) was calculated as follows[1,2]:

$$TL = 20\log_{10}\left(\frac{V_{\mathrm{RMS,withglass}}}{V_{\mathrm{RMS,noglass}}}\right)$$

The experimental data demonstrates that the transmission loss introduced by the rigid glass substrate is only -1.24 dB, retaining approximately 86.7% of the acoustic pressure amplitude.

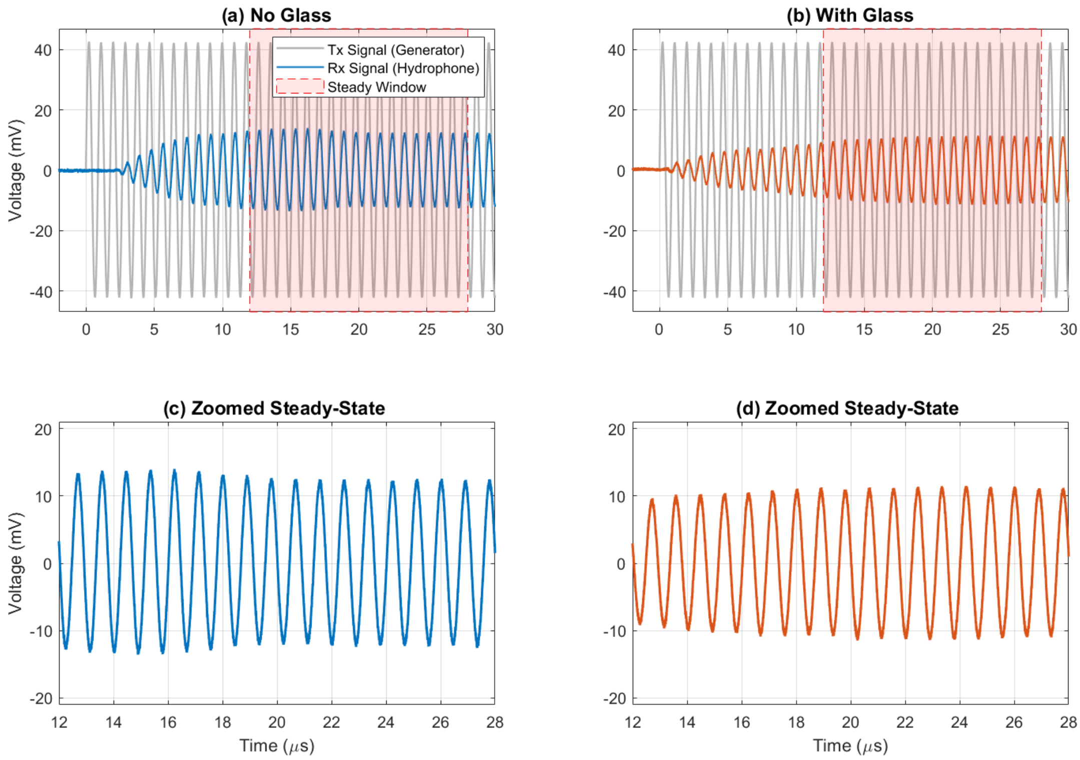

Figure S2. Acoustic transmission measurement of the vessel phantom. Acoustic waveform measured beneath the PDMS channel without the glass bottom (a) or with the glass bottom (b). (c, d) Corresponding zoomed-in views of the steady-state regions in (a) and (b), respectively.

2. Acoustic Field Mapping of the Transducer for Transwell Setup

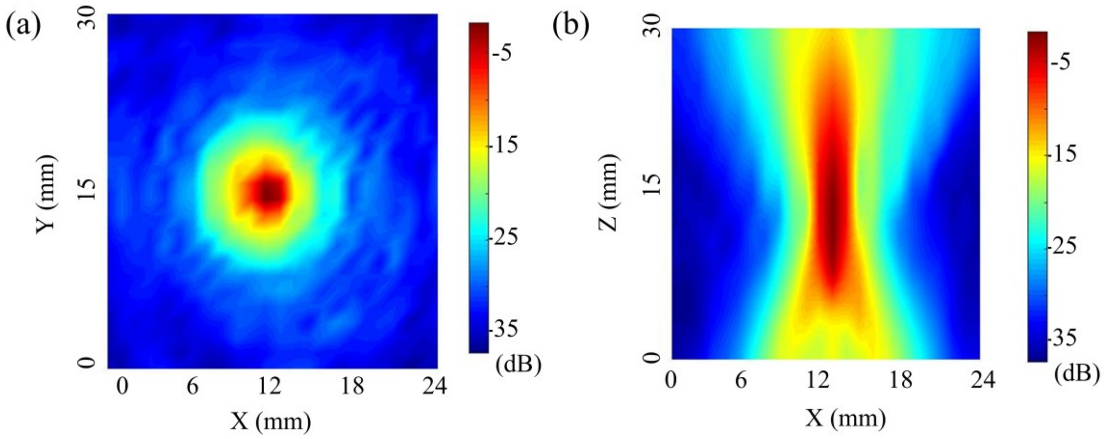

Figure S3. Acoustic field characterization of the focused ultrasound transducer utilized in the transwell setup. (a) Acoustic pressure distribution mapped in the transverse (xy) plane. (b) Acoustic pressure distribution mapped in the axial (xz) plane.

To ensure the consistency of acoustic exposure between the *in vitro* transwell assays and vessel-mimicking phantom, the acoustic fields for both experimental setups were measured. **Figure S3** displays the spatial acoustic field mapping (both xy-transverse and xz-axial focal planes) of the transducer used in the transwell setup. The driving voltage for both transducers was calibrated to ensure that the peak negative pressures (PNP) at the target regions were matched at the designated levels (e.g., 0.25 MPa and 0.5 MPa). Furthermore, the focal spot sizes for both transducers were sufficiently large to uniformly cover the regions of interest. Therefore, despite the use of different transducers to accommodate the geometry of the setups, the localized acoustic exposure and mechanical stimuli experienced by the samples were rigorously matched and highly consistent.

3. Calculation of Mechanical Index and Integrated Acoustic Energy

The Mechanical Index (MI), which dictates the instantaneous maximum amplitude of microbubble oscillation and the likelihood of inertial cavitation, is defined by the peak negative pressure (PNP) and the center frequency (f_c) [3,4]:

$$MI = \frac{\mathrm{PNP}}{\sqrt{f_c}}$$

In our experiments, the driving frequency is constant at 1.125 MHz. Therefore, for a given acoustic pressure, the MI remains identical regardless of the pulse duration: MI ≈ 0.47 at 0.5 MPa, and MI ≈ 0.24 at 0.25 MPa. This confirms that at any matched pressure level, the instantaneous mechanical stress exerted by the microbubbles is comparable between the Short Pulse (SP) and Long Pulse (LP) conditions. Both MI values fall within (MI 0.4-0.7) or below (MI<0.4) the stable cavitation range, consistent with the Passive Cavitation Detection (PCD) data measured (section 4) showing only harmonic emissions (stable cavitation) and no broadband noise (inertial cavitation).

- MI < 0.4: Generally considered safe for diagnostic ultrasound without significant cavitation risk.
- MI 0.4–0.7: Transitional range where stable cavitation may occur; inertial cavitation becomes increasingly possible.

However, because acoustic energy is proportional to the square of the pressure multiplied by the effective exposure time, the LP condition (90.9 ms effective exposure time) inherently delivers approximately 22.7 times more total integrated acoustic energy than the SP condition (4 ms effective exposure). To precisely address the integrated ultrasound energy, we calculated the time-integrated energy dose (E), assuming an acoustic impedance of Z ≈ 1.5 MRayl (here, Rayl=1Pa·s/m) for the 1X PBS medium. The spatial peak pulse average intensity (I_{SPPA}) is derived from the peak negative pressure (P), and the total integrated energy is calculated as follows:

$$E = I_{\text{SPPA}} \times t_{\text{effective}} = \left(\frac{P^2}{2Z}\right) \times t_{\text{effective}}$$

The integrated acoustic energy is approximately 0.033 J/cm^2 for the SP condition and 0.757 J/cm^2 for the LP condition at 0.5 MPa. At 0.25 MPa, the integrated acoustic energy is approximately 0.008 J/cm^2 for the SP condition and 0.189 J/cm^2 for the LP condition

Condition	Pressure (P)	$t_{\text{effective}}$ (ms)	I_{SPPA}(W/cm^2)	Integrated energy (E)
10s LP	0.5 MPa	90.9	8.333	0.757 J/cm^2
10s SP	0.5 MPa	4.0	8.333	0.033 J/cm^2
10s LP	0.25 MPa	90.9	2.083	0.189 J/cm^2
10s SP	0.25 MPa	4.0	2.083	0.008 J/cm^2

The PCD spectra (**Figure S4b, Figure S5b**) reveal that the instantaneous harmonic emission intensities (e.g., $2f_0$, $3f_0$) are highly comparable between the SP and LP conditions, further corroborating the identical MI.

4. Passive Cavitation Detection (PCD) and Cavitation Regime Analysis

To define the exact cavitation regime and rule out harmonic generation from nonlinear acoustic propagation, PCD measurements were conducted utilizing a degassed water control (without microbubbles). To guarantee experimental reproducibility and statistical reliability, the PCD measurements for each acoustic condition (including both water controls and microbubble groups) were independently repeated six times. The acoustic emission signatures remained highly consistent across all replicates, and representative data are presented herein.

At the maximum pressure of 0.5 MPa, the water controls exhibit the fundamental frequency (f_0) with a flat baseline (**Figure S4a, Figure S5a**). In contrast, under both SP and LP conditions, the microbubble groups exhibit higher harmonic emissions (e.g., $2f_0$, $3f_0$) (**Figure S4b, Figure S5b**). This provides direct acoustic evidence that microbubbles undergo stable cavitation and the systems are operating below the inertial cavitation threshold, even at our highest acoustic pressure of 0.5 MPa.

Furthermore, since the SP condition involves intermittent sonication, it is critical to confirm that microbubbles are not rapidly destroyed after the initial acoustic cycles. As explicitly detailed in the zoomed-in panels of **Figure S4** and **Figure S6** (capturing individual bursts at 0 ms, 4 ms, and 9 ms), the microbubbles consistently exhibit identical stable cavitation spectra without any stochastic broadband noise associated with inertial collapse. This confirms the temporal durability and pulse-to-pulse consistency of the stable cavitation regime across the entire stimulation window. Finally, similar stable cavitation signatures, characterized by ultra-harmonic emissions (e.g., $2f_0$, $3f_0$), were also confirmed at the lower acoustic pressure of 0.25 MPa for both SP and LP conditions (**Figure S6, Figure S7**).

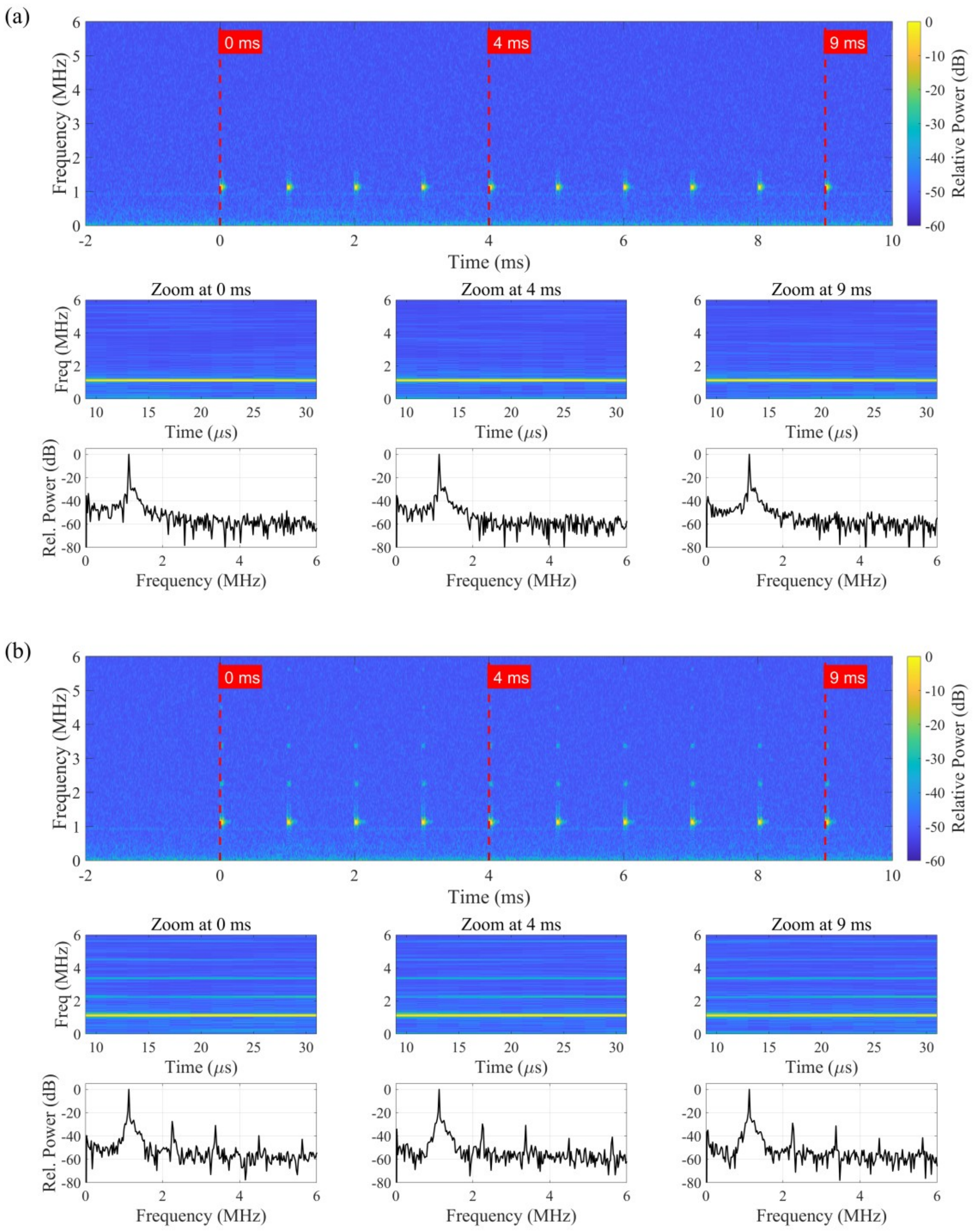

Figure S4. Passive Cavitation Detection (PCD) measurement and analysis for short pulse at acoustic pressure of 0.5 MPa. (a) Degassed water control. (b) Microbubble condition. The hydrophone was placed beneath the middle of the central channel.

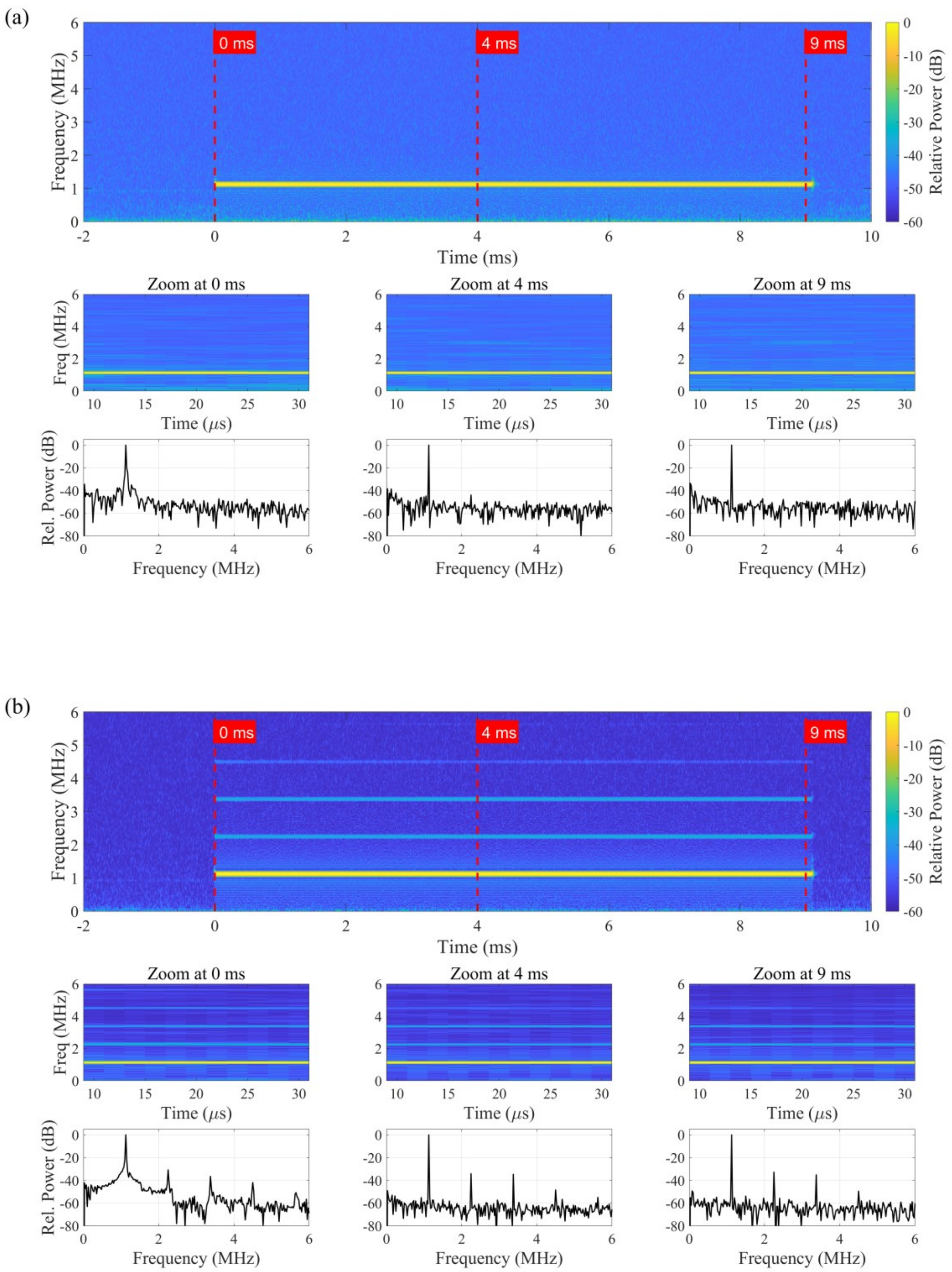

Figure S5. Passive Cavitation Detection (PCD) measurement and analysis for long pulse condition at acoustic pressure of 0.5 MPa. (a) Degassed water control. (b) Microbubble condition.

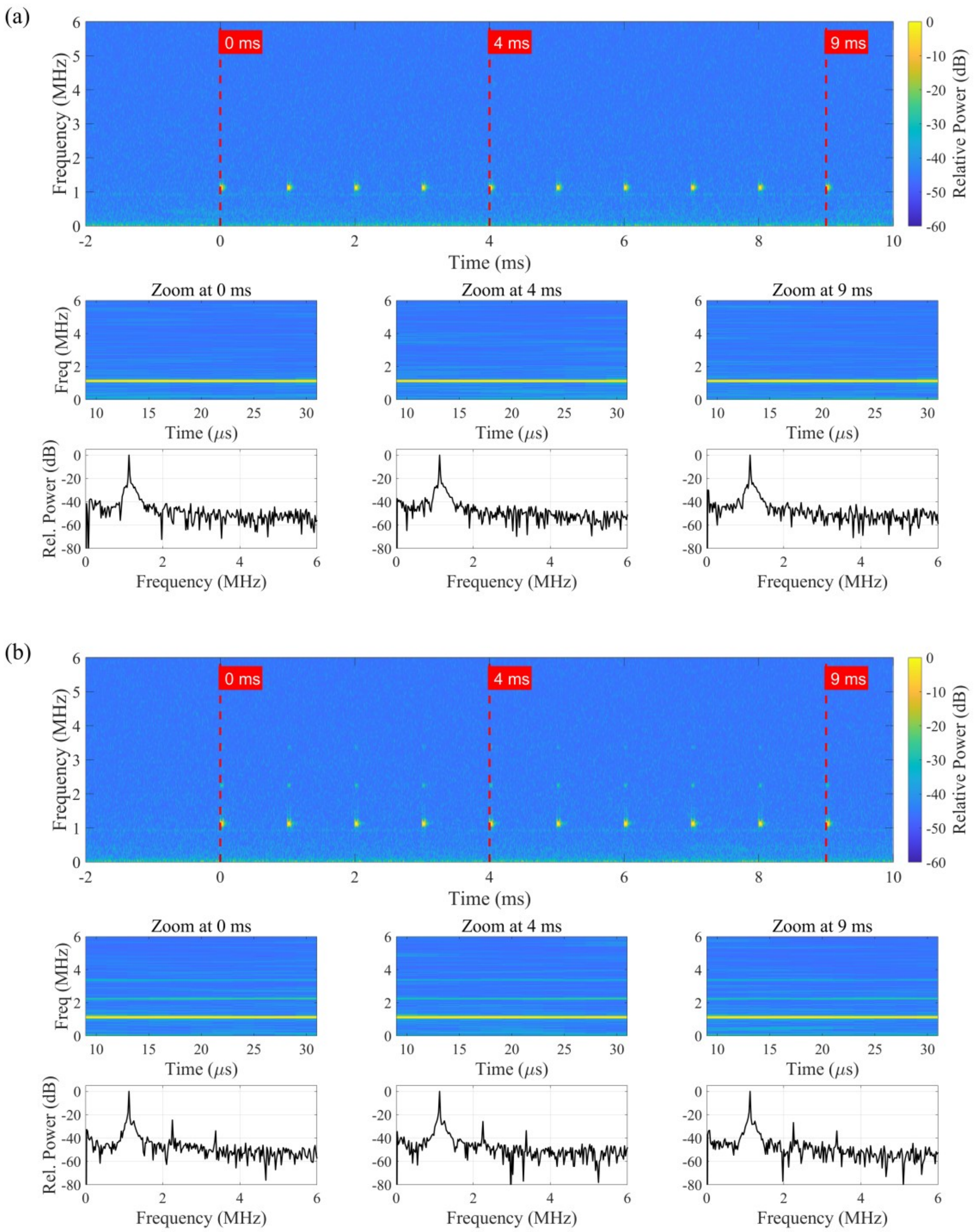

Figure S6. Passive Cavitation Detection (PCD) measurement and analysis for short pulse condition at acoustic pressure of 0.25 MPa. (a) Degassed water control. (b) Microbubble condition. The hydrophone was placed beneath the middle of the central channel.

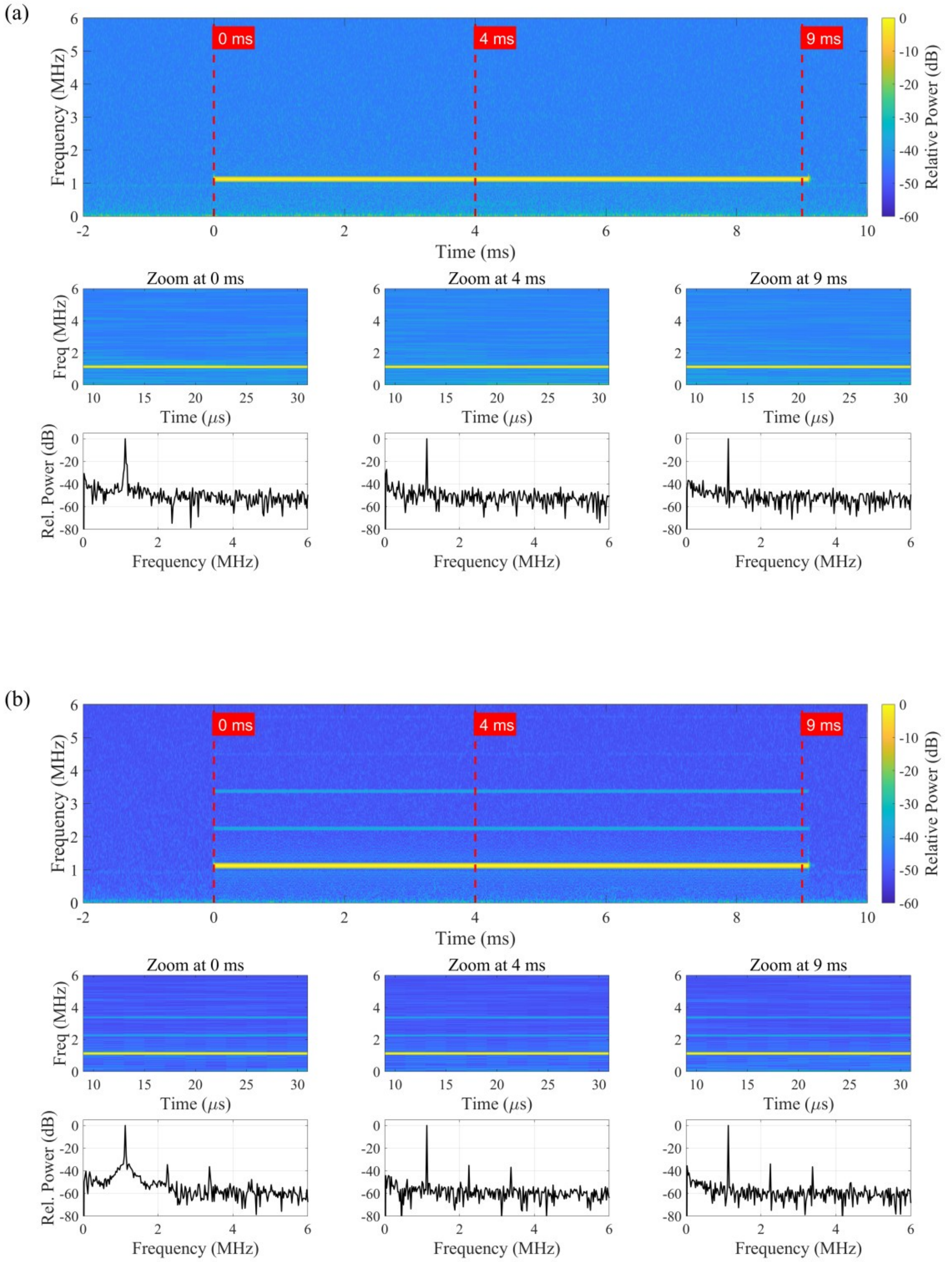

Figure S7. Passive Cavitation Detection (PCD) measurement and analysis for long pulse condition at acoustic pressure of 0.25 MPa. (a) Degassed water control. (b) Microbubble condition.

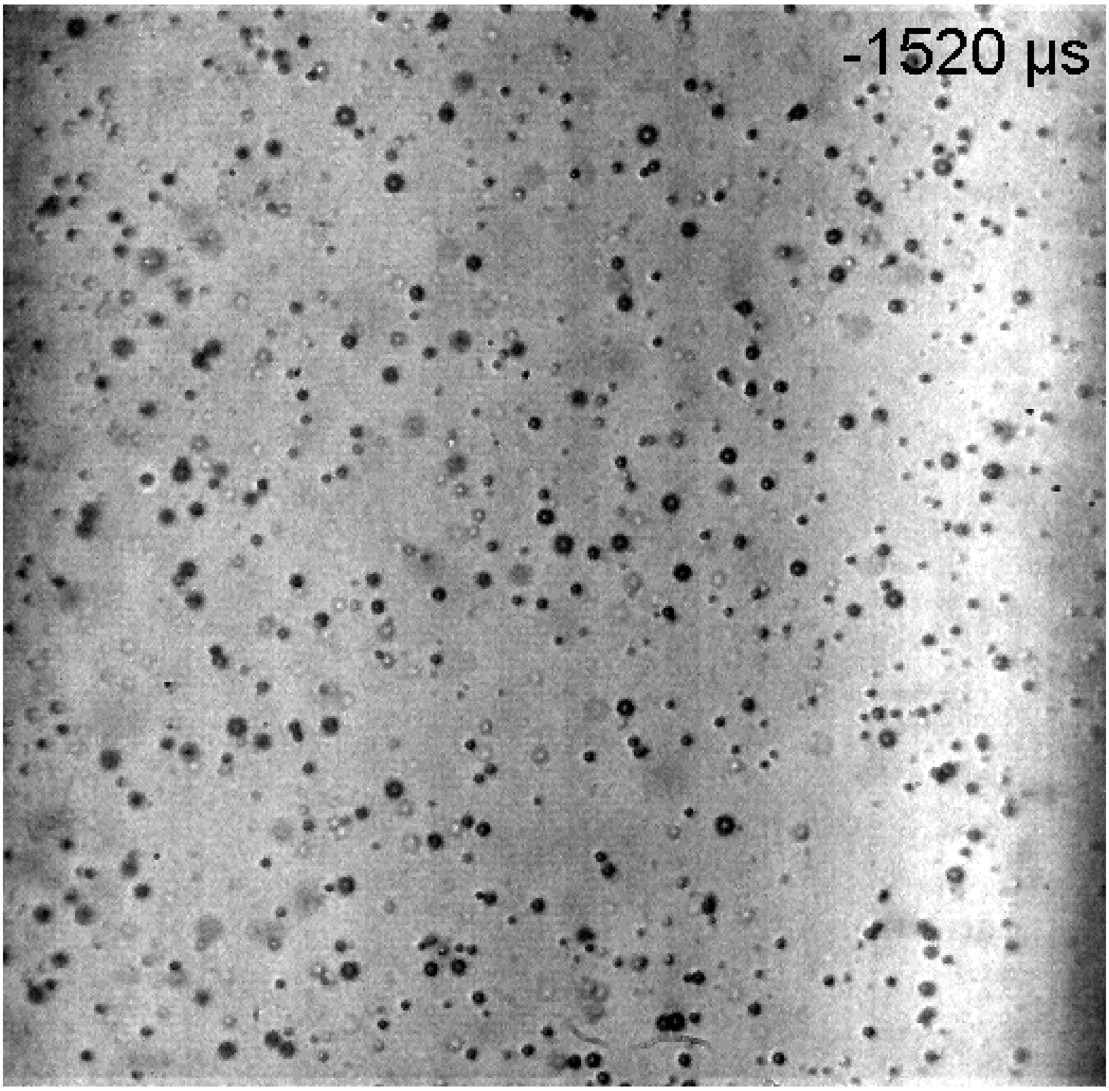

Movie S1. Representative bubble dynamics recorded under short pulse ultrasound of 0.25 MPa. Here ultrasound is on between 0-40 (1000-1040, 2000-2040, etc.) μs. Displacement of microbubbles is observed due to acoustic radiation force and mild bubble coalescence occurs due to the secondary Bjerkness force.

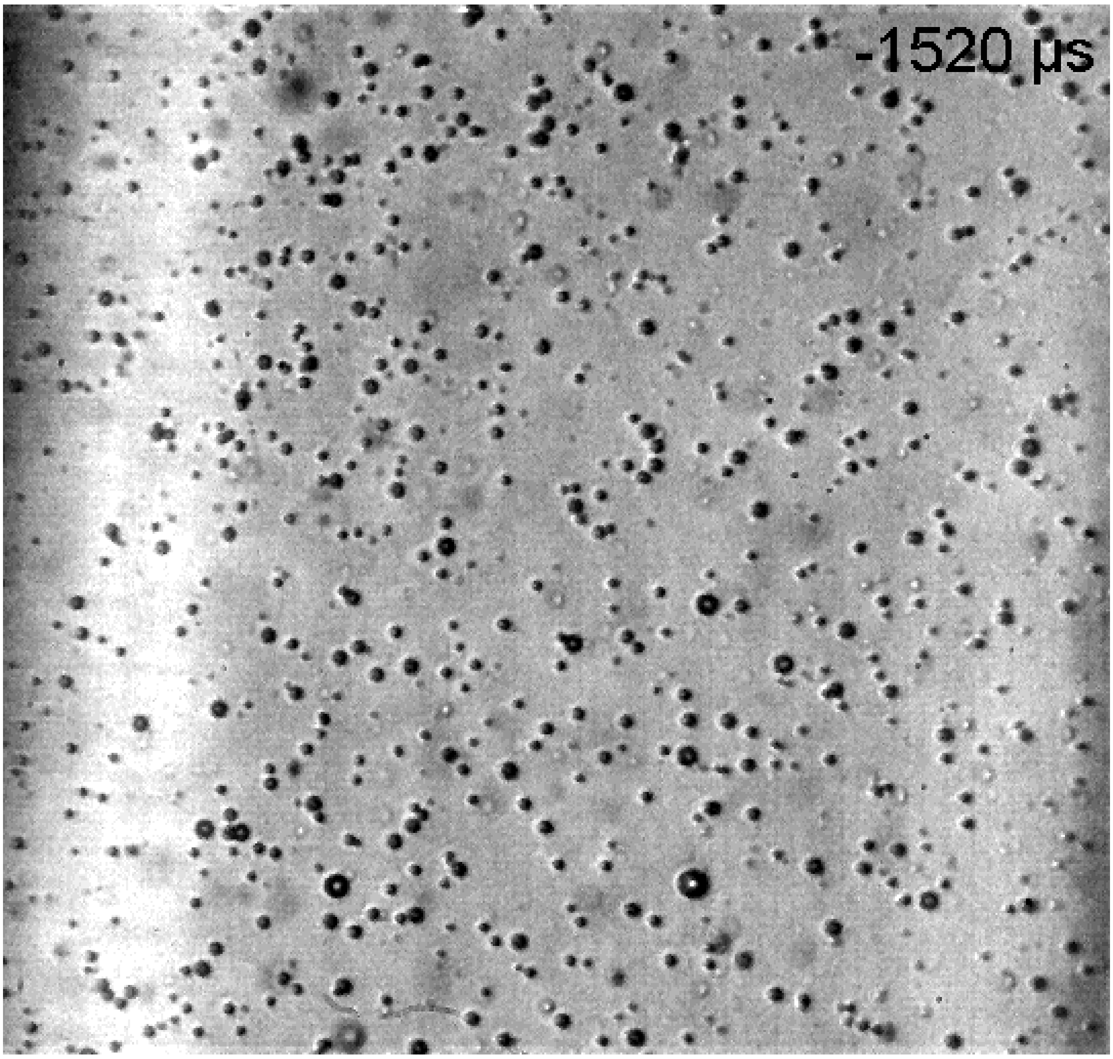

Movie S2. Representative bubble dynamics recorded under short pulse ultrasound at 0.50 MPa. Here ultrasound is on between 0-40 (1000-1040, 2000-2040, etc.) μs. Displacement and coalescence of microbubbles become more evident, resulting in larger maximum bubble size and reduced number of bubbles.

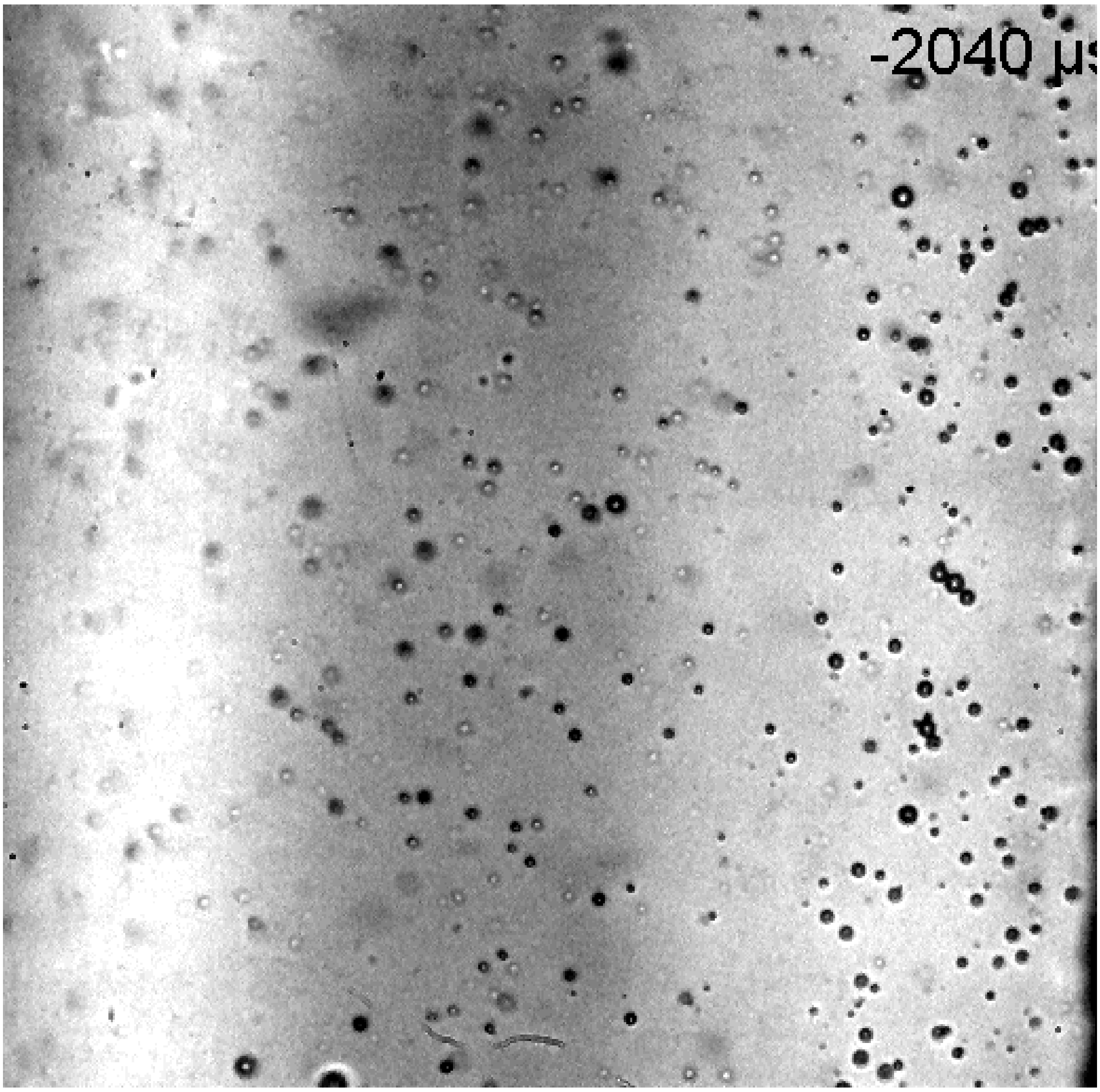

Movie S3. Representative bubble dynamics recorded under long pulse ultrasound at 0.25 MPa acoustic pressure. Here ultrasound is on between 0-9090 μs. Displacement and clustering of microbubbles are observed.

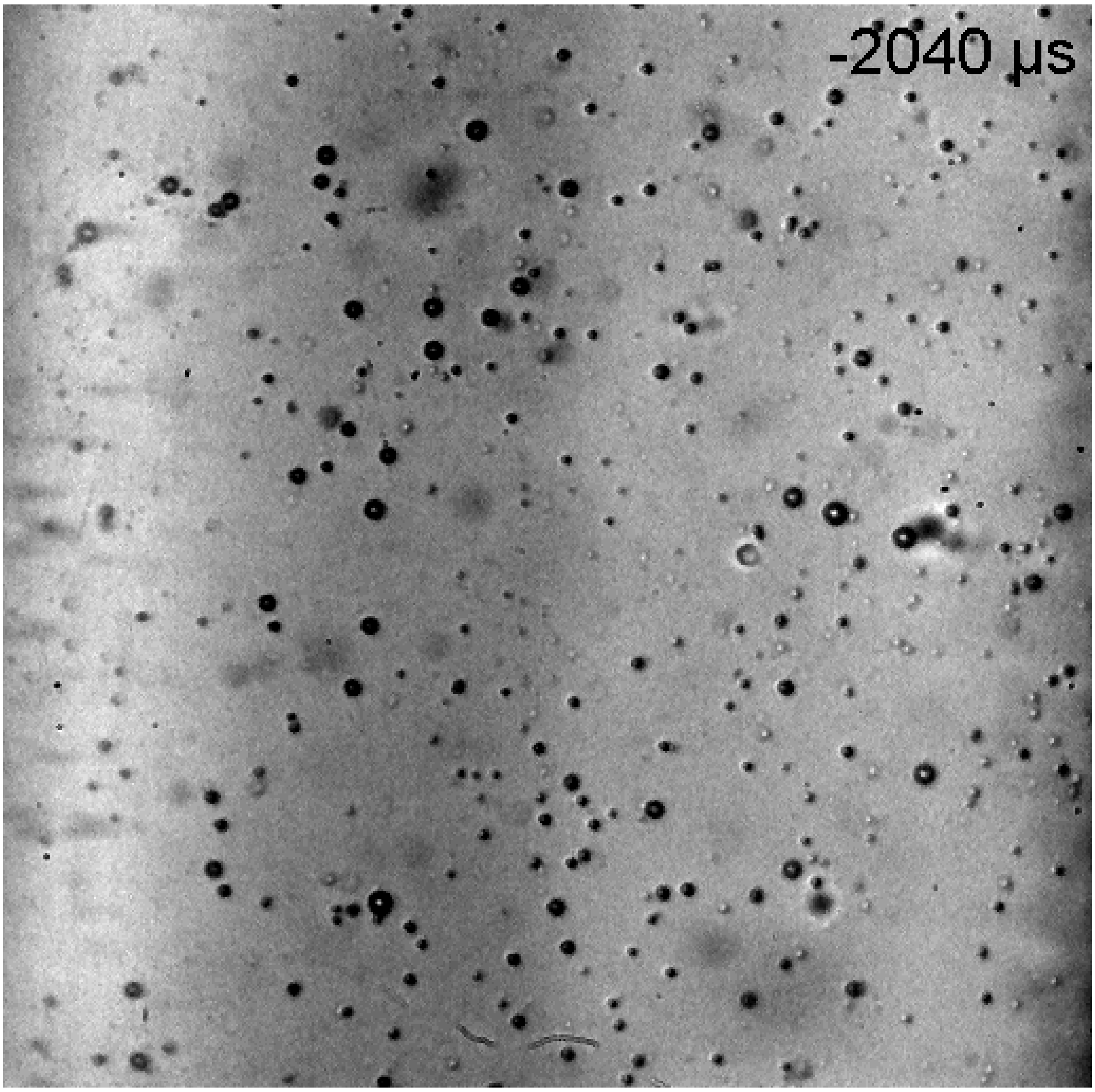

Movie S4. Representative bubble dynamics recorded under long pulse ultrasound at 0.50 MPa acoustic pressure. Here ultrasound is on between 0-9090 µs. The displacement and coalescence of microbubbles were more pronounced, stable cavitation effects occurred in the later stage with bubble oscillation.

References

1 Secomski, W. *et al.* In vitro ultrasound experiments: Standing wave and multiple reflections influence on the outcome. *Ultrasonics* **77**, 203-213, doi:10.1016/j.ultras.2017.02.008 (2017).

2 Szabo, T. L. (2014). Diagnostic Ultrasound Imaging: Inside Out (2nd ed.). Academic Press, pp. 82-83.

3 Apfel, R. E. & Holland, C. K. Gauging the likelihood of cavitation from short-pulse, low-duty cycle diagnostic ultrasound. *Ultrasound Med Biol* **17**, 179-185, doi:10.1016/0301-5629(91)90125-g (1991).

4 Abbott, J. G. Rationale and derivation of MI and TI--a review. *Ultrasound Med Biol* **25**, 431-441, doi:10.1016/s0301-5629(98)00172-0 (1999).
