# Supplementary material for: Tuning endothelial barrier permeability with ultrasound: a pulse-length-dependent interplay between bubble dynamics and cellular bioeffects
Source: Ultrason Sonochem. 2026 Apr 12;129:107851. doi: 10.1016/j.ultsonch.2026.107851 (PMC13139967; doi:10.1016/j.ultsonch.2026.107851)
Supplement: Supplementary Data 2 [file mmc2.docx]

Legends for Figs. S1 to S7

Legends for Movies S1 to S4

**Figure S1. Schematics of the experimental setup for acoustic measurement.**

**Figure S2.** **Acoustic transmission measurement of the vessel phantom**. Acoustic waveform measured beneath the PDMS channel without the glass bottom (a) or with the glass bottom (b). (c, d) Corresponding zoomed-in views of the steady-state regions in (a) and (b), respectively.

**Figure S3. Acoustic field characterization of the focused ultrasound transducer utilized in the transwell setup.** (a) Acoustic pressure distribution mapped in the transverse (xy) plane. (b) Acoustic pressure distribution mapped in the axial (xz) plane.

| Condition | Pressure ($P$) | $t_{\mathrm{effective}}$ (ms) | $I_{\mathrm{SPPA}}$(W/cm^2^) | Integrated energy (E) |
| --- | --- | --- | --- | --- |
| 10s LP | 0.5 MPa | 90.9 | 8.333 | 0.757 J/cm² |
| 10s SP | 0.5 MPa | 4.0 | 8.333 | 0.033 J/cm² |
| 10s LP | 0.25 MPa | 90.9 | 2.083 | 0.189 J/cm² |
| 10s SP | 0.25 MPa | 4.0 | 2.083 | 0.008 J/cm² |

**Table S1. Effective ultrasound exposure time, acoustic intensity and integrated energy for 10 s short and long pulse at different acoustic pressures.**

**Figure S4.** Passive Cavitation Detection (PCD) measurement and analysis for short pulse at acoustic pressure of 0.5 MPa. (a) Degassed water control. (b) Microbubble condition. The hydrophone was placed beneath the middle of the central channel.

**Figure S5.** Passive Cavitation Detection (PCD) measurement and analysis for long pulse condition at acoustic pressure of 0.5 MPa. (a) Degassed water control. (b) Microbubble condition.

**Figure S6.** Passive Cavitation Detection (PCD) measurement and analysis for short pulse condition at acoustic pressure of 0.25 MPa. (a) Degassed water control. (b) Microbubble condition. The hydrophone was placed beneath the middle of the central channel.

**Figure S7.** Passive Cavitation Detection (PCD) measurement and analysis for long pulse condition at acoustic pressure of 0.25 MPa. (a) Degassed water control. (b) Microbubble condition.

**Movie S1.** **Representative bubble dynamics recorded under short pulse ultrasound of 0.25 MPa**. Here ultrasound is on between 0-40 (1000-1040, 2000-2040, etc.) µs. Displacement of microbubbles is observed due to acoustic radiation force and mild bubble coalescence occurs due to the secondary Bjerkness force.

**Movie S2. Representative bubble dynamics recorded under short pulse ultrasound at 0.50 MPa.** Here ultrasound is on between 0-40 (1000-1040, 2000-2040, etc.) µs. Displacement and coalescence of microbubbles become more evident, resulting in larger maximum bubble size and reduced number of bubbles.

**Movie S3. Representative bubble dynamics recorded under long pulse ultrasound at 0.25 MPa acoustic pressure**. Here ultrasound is on between 0-9090 µs. Displacement and clustering of microbubbles are observed.

**Movie S4. Representative bubble dynamics recorded under long pulse ultrasound at 0.50 MPa acoustic pressure.** Here ultrasound is on between 0-9090 µs. The displacement and coalescence of microbubbles were more pronounced, stable cavitation effects occurred in the later stage with bubble oscillation.
